# Seasonal dynamics of spatial distributions and overlap between Northeast Arctic cod (*Gadus morhua*) and capelin (*Mallotus villosus*) in the Barents Sea

**DOI:** 10.1371/journal.pone.0205921

**Published:** 2018-10-16

**Authors:** Johanna Fall, Lorenzo Ciannelli, Georg Skaret, Edda Johannesen

**Affiliations:** 1 Institute of Marine Research, Bergen, Norway; 2 College of Earth, Ocean and Atmospheric Sciences, Oregon State University, Corvallis, Oregon, United States of America; Hellenic Centre for Marine Research, GREECE

## Abstract

The trophic link between cod (*Gadus* sp.) and capelin (*Mallotus sp*.) is important in many panarctic ecosystems. Since the early 2000s, the Northeast Arctic cod stock (*G*. *morhua*) in the Barents Sea has increased greatly, and the sea has been exceptionally warm. Such changes have potentially large effects on species distributions and overlap, which in turn could affect the strength of species interactions. Due to its high latitude location, the Barents Sea has strong seasonal variation in physical conditions and interactions. To study drivers of variation in cod-capelin overlap, we use data from two annual surveys run in winter and in autumn of 2004–2015. We first model winter and autumn spatial distributions of mature and immature cod and capelin. We then calculate overlap from model predictions on a grid with similar spatial resolution as the survey data. Our approach allowed us to interpret changes in overlap as species-specific effects of stock size and temperature, while accounting for sampling variation due to sampling time and depth. We found that during winter both species expanded their distribution in response to increased stock sizes, but how strongly and where the expansion occurred varied. The effect of temperature on distributions varied in space, and differed for cod and capelin and for different components of the two species. The results for autumn were clearer and more consistent. Both species expanded their distribution areas as their stock sizes increased. A positive effect of temperature was found in the north-eastern Barents Sea, where temperatures were lowest at the start of the study. Overlap increased and shifted north-eastwards during the study period and remained high despite a decline in the capelin stock. The increased overlap during autumn could mainly be attributed to the shift in cod distribution with increased cod stock biomass.

## Introduction

Spatial association or *overlap* between predator and prey is a prerequisite for predation to take place. Understanding the drivers of overlap is thus underlying any assessment of predation rate and natural mortality of a prey. In a fishery context, overlap has potentially important implications for management because of its influence on stock dynamics [1]. A strong overlap giving a positive linear relationship between predator and prey densities across space is expected if a predator perfectly tracks its prey [2–4]. However, both predators and prey are influenced by other factors that vary in space, such as interaction with other species and physical properties of the environment. These factors may impose constraints on behaviour and distributions, creating non-linear and spatially varying relationships between predators and prey. Spatially explicit analyses, where species distributions are evaluated for given geographic locations in a heterogenous landscape [5], are therefore more appropriate than aggregating across space for understanding factors underlying changes in predator-prey overlap over time [6, 7].

In several shelf ecosystems in the panarctic region, cod (*Gadus* sp.) and capelin (*Mallotus* sp.) are abundant species forming an important predator-prey interaction [8]. In the Barents Sea, too, the trophic link between the commercially important stocks of Northeast Arctic cod (*Gadus morhua*; hereafter cod) and Barents Sea capelin (*Mallotus villosus*; hereafter capelin) is key for the ecosystem dynamics. Cod is the main predator on post-larval capelin [9–11], and although cod is a generalist, it has an apparent preference for capelin [10, 12, 13]. The spatial distributions and life cycles of both species are adapted to the strong seasonality in this high latitude ecosystem. The northern Barents Sea is seasonally ice-covered, and here the spring bloom after ice melt supports a rich zooplankton production [14]. Capelin migrate northwards to feed on the zooplankton, followed by cod [13]. The main feeding season lasts throughout the summer into early autumn, after which cod and capelin shift further south. Both species spawn in early spring; capelin spawns along the northern coast of Norway and Russia, while cod’s main spawning ground is further south along the Norwegian coast in the Lofoten area [13]. As a consequence, the overlap and interaction between the species vary seasonally; from diet data, it appears that cod’s preference for capelin is stronger during winter than in summer [15].

During the past ten years, the cod stock has increased to similar levels as in the late 1940s, when abundance had increased in the absence of fishing during World War II [16]. Concurrent with the increase in stock size, cod has expanded northwards both in winter and in the late summer/early autumn feeding season [16–18], potentially affecting the cod-capelin overlap. The Barents Sea capelin stock is known for strong fluctuations in abundance, resulting in a pattern of stock collapses and recoveries [19]. Currently, the stock is recovering from a collapse [20]. While the fishery is closed during stock collapses, mature capelin is subject to commercial harvesting in periods of high abundance. The stock assessment of capelin was among the first to extend beyond single-stock evaluation by explicitly modelling effects of the cod stock on capelin mortality in the stock projection simulation [21–23]. The stock assessment model relies on several assumptions related to the seasonal interaction between cod and capelin [24], but recent changes in seasonal cod distribution and feeding have not been incorporated [15, 16, 19]. For a long time, it has also been an unachieved objective to include spatially explicit information about the cod-capelin interaction in the model [24].

Based on cod stomach data and a large body of work describing seasonal distributions and migration patterns of cod and capelin ([9, 25], and references therein), the overlap between the species has been inferred, but not studied directly. Furthermore, overlap metrics and robust statistical methods for predicting overlap have not been established.

**Fig 1:**
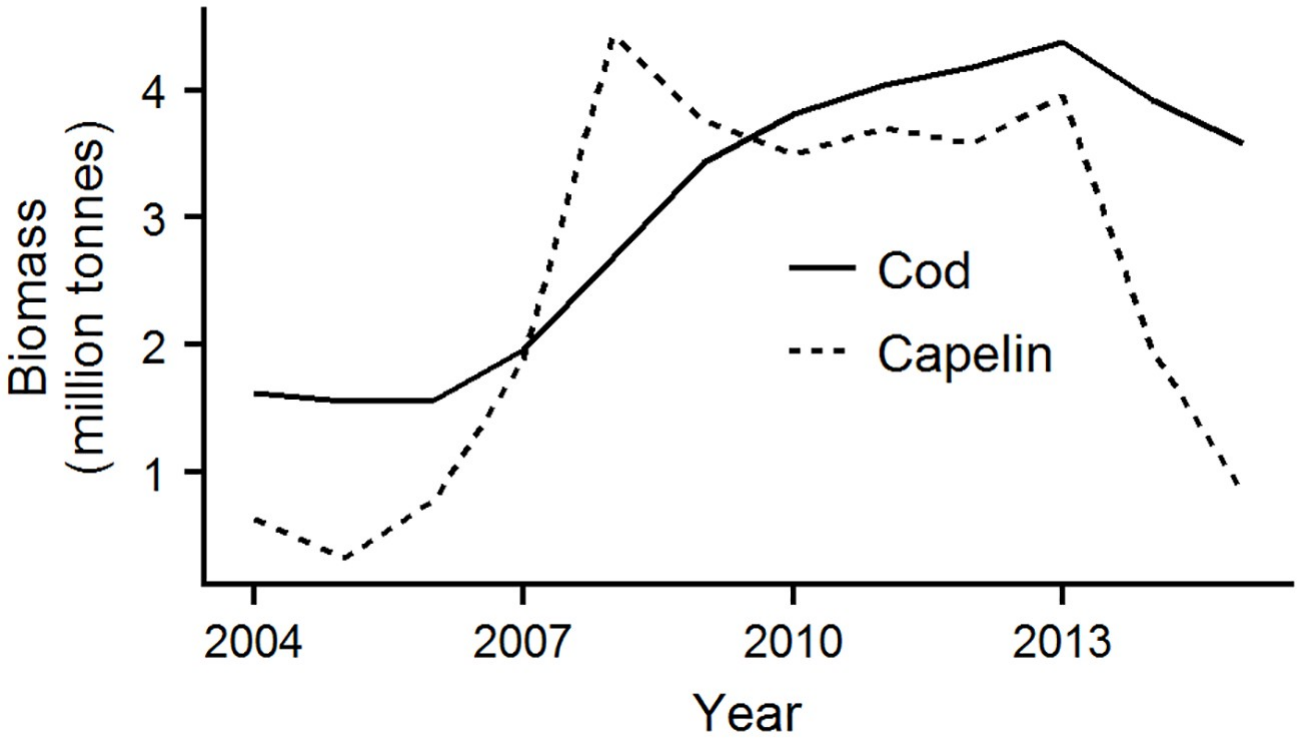
Cod and capelin stock biomass. Biomass of cod (age 3+, estimated in winter) and capelin (age 1+, estimated in autumn) in the study years 2004–2015. The capelin biomass is from the assessment based on the acoustic estimate from the ecosystem survey, and the cod biomass is the most recent published stock assessment (cod 3+, capelin 1+, Tables 3.18 and 9.4 in [27]).

Here, we study seasonal and spatial aspects of cod-capelin overlap from 2004–2015, covering a period with exceptionally high water temperatures [26], two capelin collapses and a more than doubling of cod biomass (Fig 1). We address the need for new knowledge and improved methods for appraising cod-capelin spatial overlap through 1. Examining how cod and capelin distributions in late summer and winter relate to temperature and stock biomass using spatially explicit modelling tools, 2. Developing an index of spatial overlap, and assessing cod-capelin overlap in each season during the study period, and 3. Discussing how variation in the overlap across the study period relates to the factors identified in 1.

## Methods

### Study area and data collection

**Fig 2:**
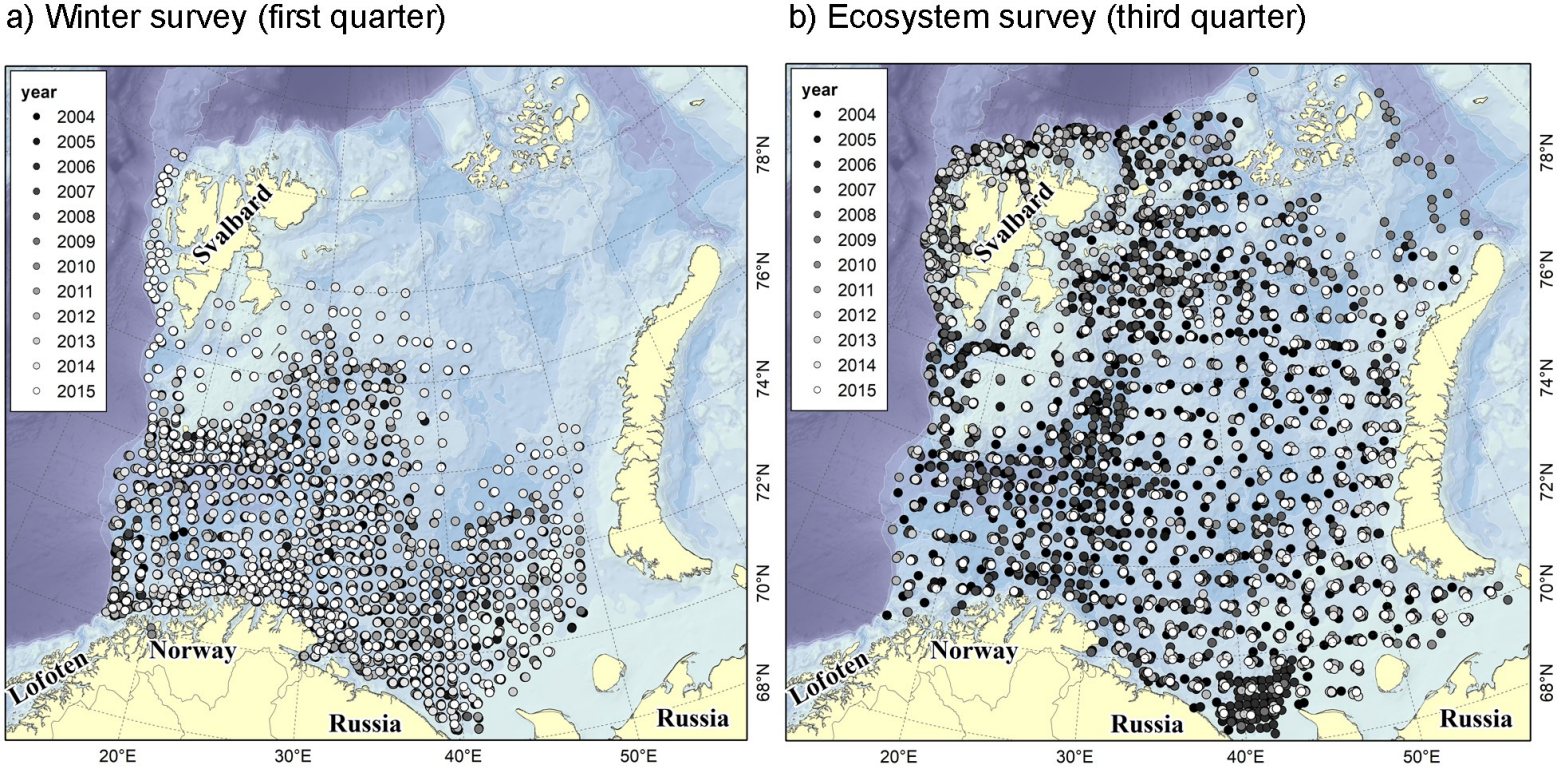
Study area and sampling stations. Demersal trawl stations used in the present study from A) the winter survey and B) the ecosystem survey in 2004–2015. The shade of the points indicates if the station was sampled early (dark) or late (light) in the study period. The background highlights the main bathymetric features of the Barents Sea. The winter survey runs in January—March each year with the purpose of obtaining abundance indices for stock assessment of cod and haddock (*Melanogrammus aeglefinus*). The winter survey has a stratified regular design with higher station density in strata with historically higher abundance of cod to minimize the overall sampling variance in the cod estimates. The ecosystem survey covers most of the Barents Sea shelf in August to early October. The aim of the survey is firstly to provide an acoustic estimate of the capelin stock for assessment and quota advice, and secondly to assess the ecosystem state by monitoring the most important ecosystem components. The ecosystem survey has a regular sampling grid, but higher station density around Svalbard due to strong depth gradients in this area, in the Hopen trench (2004–2007) due to higher densities of *Pandalus borealis*, and east of Svalbard due to higher density of capelin. In 2014, unusual ice conditions restricted the coverage of the northern Barents Sea in the ecosystem survey.

The Barents Sea is a high latitude shelf sea bordering the polar basin to the north and the coasts of Russia and Norway to the south (Fig 2). Two Norwegian-Russian surveys with comprehensive coverage are conducted annually in the Barents Sea: the *winter survey* (1981 –) covering the south-central Barents Sea in the pre-spawning season of cod and capelin when both species undertake their spawning migration (Fig 2A), and the *ecosystem survey* (2004 –) covering the whole shelf in the main feeding season (Fig 2B). To be able to compare the two seasons, only data collected in the period 2004–2015 were used here. Data from the Norwegian surveys are available from the Dryad Digital Repository: https://doi.org/10.5061/dryad.pv3rc1m.

Both surveys use a Campelen demersal shrimp trawl at fixed stations for near-bottom sampling as a basis for swept area abundance estimation. The interstation distance has ranged between 15 and 35 nautical miles (nmi) (28–65 km, Fig 2). All vessels have been equipped with Simrad EK60 echo sounders (on some vessels Simrad EK500 during the first years) for recording and integrating fish echoes along the survey tracks. The acoustic backscatter is allocated to target groups based on species-specific acoustic properties and the catch composition in pelagic and bottom trawls, and then integrated over a horizontal distance of 5 nmi (9.3 km, 2004–2007) or 1 nmi (1.9 km, 2008–2015). The most important biological data support for the pelagic acoustic data interpretation comes from “Harstad trawl” samples [28], which provide data both from fixed stations and from sampling of specific acoustic recordings for validation. CTD casts for temperature measurements are made in conjunction with trawl tows, and depth at the start of the tow is recorded by Scanmar trawl sensors (for more details about the two surveys, see [29] and [30]).

Cod densities (number of individuals/nmi^2^) were estimated using standard methods for cod swept area calculation in the Barents Sea, that is, number caught at each trawl station divided by trawled area, assuming that the effective fishing width along the trawled transect is dependent on cod length [30]. The standard trawled distance was 1.5 nmi (2.8 km) for the winter survey in 2004–2010, and 0.75 nmi (1.4 km) for the entire autumn survey and the winter survey after 2010. Since immature and mature cod have different distributions, particularly in winter [31], we divided the swept area density estimates into one immature and one mature cod component, using the average age at 50% maturity for the study years and length at age data from the surveys (winter: mature cod ≥ 70 cm, autumn: mature cod ≥ 75 cm, [27]).

Autumn capelin acoustic densities (in units of Nautical Area Scattering Coefficient; NASC; m^2^/nmi^2^) were based on data collected during the ecosystem survey, the same data which is used to provide an absolute abundance estimate for the capelin stock assessment each year [27]. The acoustic data from winter is based on the same methodology, but is of lower quality as this survey mainly targets demersal fish and has few pelagic trawl hauls for acoustic target verification (on average 6 hauls versus 38 for the ecosystem survey). The winter survey also coincides with the period when the mature part of the capelin stock is undertaking its spawning migration, and capelin seems to be less available to acoustic detection during spawning migration than at other times [32, 33]. We therefore chose to supplement the winter acoustic data with density estimates of capelin from the demersal trawl (number of individuals/nmi towed), and hereafter refer to capelin from the different sampling methods as “acoustic capelin” and “trawl capelin”, respectively.

### Data preparation and analysis

We first developed single species distribution models for cod and capelin for autumn and winter using Generalised additive models (GAM, [34]) (*Single species distribution models* below), and then calculated overlap from model predictions of local species densities (*Quantifying overlap* below). We chose to do this rather than calculate overlap directly from the raw data, since we wished to relate changes in overlap to *species-specific* responses to the environment. In addition, our model predictions included variables controlling for sampling variation that could have biased indices calculated on raw data [35].

#### Single-species distribution models

As density-independent predictors in the species distribution models, we used water temperature (bottom temperature for cod, and mean pelagic temperature from 50–200 m depth for capelin), bottom depth, sun height, survey day, and year. The temperature variables were allocated to each trawl station from the nearest CTD measurement from the same survey. The bottom depth was that measured at the beginning of trawling. Sun height was included to account for sampling variation associated with diurnal vertical migrations, and was calculated from the day of the year, geographical position, and sampling time. Survey day was expressed relative to the earliest day of the season (autumn, winter) across the study period when sampling took place, and was included to account for the quasi-synoptic coverage and inter-annual differences in timing of sampling in relation to the migrations of cod and capelin. The geographical coordinates *x*,*y* were projected stereographically with centre in the middle of our study area at 75° N and 35° E, and expressed in deviation from this centre in nautical miles. Finally, we included the annual total stock biomasses [27] of each species as covariates to test for potential density-dependent effects on species distributions. As the capelin stock assessment is done on data from the autumn survey, we used the capelin stock biomass from the previous year in the winter models of capelin distribution.

All data points containing missing values in any covariate were removed. To avoid large outliers in the covariates, we also limited the data to include bottom temperatures in the range -2 to +7.5°C, and depths of 50–500 m, which contained the bulk of observations in both seasons, leaving 3994 observations for analysis in winter, and 4644 observations in autumn. Calculation of variance inflation factors indicated that the correlations between covariates were not a cause for concern (values < 3, [36]), except for the correlation between year and stock biomasses. We therefore used stock biomass only.

Since the Barents Sea contains many islands and complex coastlines, we applied the soap film smoother in the GAMs (for details, see S1 Appendix). All analyses were done in R version 3.4.1 for Windows [37], using the packages *mgcv* [38, 39] for GAM fitting, and *ggplot2* [40], *cowplot* [41] and *itsadug* [42] for visualisation.

The response variables in the models were local cod and capelin densities. Due to the large amount of acoustic data and the application of the soap film smoother, convergence of the capelin models was problematic. We therefore chose to include only acoustic registrations adjacent to the bottom trawl stations, using distance weighted interpolation of the area backscatter (NASC) within a 15 nmi (28 km) radius with weights of the form *w*_*i*_ = (1 + *d*_*i*_)^-1^, where *d*_*i*_ is the Euclidian distance between the acoustic sampling points and the station [43]. This did not lead to any loss of information relevant to our objectives, as initial runs using the finer resolution data gave similar response-covariate relationships.

For each season and *component* (immature cod, mature cod, acoustic capelin, trawl capelin), we fitted separate distribution models with the untransformed species density *D*_*(x*,*y)*,*t*_ in position *x*,*y* in year *t* as the response, conditional on other environmental covariates, using a GAM with Tweedie distribution and the default log-link. The variance of the Tweedie distribution is related to the mean by a power function (Eq 1):

$$var(Y)=\mu^{p}$$ (1)

While a *p* of 0, 1 or 2 corresponds to the familiar Gaussian, Poisson and Gamma distributions, respectively, for 1 < *p* < 2, the Tweedie distribution is a compound Poisson–gamma distribution with mass at zero, appropriate for our data. This is also the range where automatic estimation of the power parameter during fitting is implemented in *mgcv* [44]. Firstly, we fit models with basic smooth term predictors to establish baselines for comparison with more complex formulations, and to evaluate the overall (across-space) relationships between covariates and response (*habitat model* Eq 2):

$$D_{(x,y),t}=\alpha +s_{1}(x,y)+s_{2}({bio}_{t})+s_{3}({depth}_{x,y})+s_{4}({sun}_{(x,y),t}){+s}_{5}({temp}_{(x,y),t})+s_{6}({s.day}_{(x,y),t})+\varepsilon_{(x,y),t}$$ (2)

Here *s*_*1-6*_ are smooth functions of geographical position, stock biomass, depth, sun height, temperature, and survey day, respectively, *α* is the global intercept and *ε*_*(x*,*y)*,*t*_ is the error term whose variance is related to the mean according to Eq 1 under the Tweedie distribution. To avoid overfitting the smooth functions, we constrained their level of wiggliness by limiting the maximum number of basis dimensions (“knots”) to 5 on the univariate smooths and 20 on the two-dimensional smooth of geographical position. Thereafter, we systematically increased model complexity, ending up with seven *candidate models* describing the distribution of each component. These models included different combinations of the covariates in Eq 2 and spatially variant terms of stock biomass, temperature, and survey day. Spatially variant terms test for linear effects of a variable, but the effect is also allowed to vary smoothly in space so that there may be a positive effect in one part of the study area, and a negative effect in another [45]. The most complex candidate models were on the form:

$$D_{(x,y),t}=\alpha +s_{1}(x,y)+s_{2}({depth}_{x,y})+s_{3}({sun}_{(x,y),t})+s_{4}(x,y)\times {temp}_{(x,y),t}+s_{5}(x,y){\times s.day}_{(x,y),t}+s_{6}(x,y)\times {bio}_{t}+\varepsilon_{(x,y),t}$$ (3)

Where each product of geographical position and a covariate represents a spatially variant term. The models contained either a regular smooth or a spatially variant term of the same covariate.

From the candidate models, one model for each component was selected for overlap calculations based on minimisation of the Akaike Information Criterion (AIC) and maximisation of deviance explained after backwards elimination of non-significant predictors. The relationships between response and covariates were assessed by examining their robustness across model formulations, i.e., if the relationships were stable or varied, where the latter could indicate that the predictor captured residual variation in the model rather than a meaningful pattern. The models were visually inspected for residual correlation using the R-functions *pacf* (temporal correlation) and *variog* (spatial correlation, library *geoR*, [46]). None of the model residuals showed temporal autocorrelation, but the residuals of the capelin models using acoustic data were spatially autocorrelated. Since this may cause an underestimation of confidence intervals, we performed a wild bootstrap [47] on the capelin habitat models. The wild bootstrap followed the same steps implemented by Llope et al. [48] to model phytoplankton distribution in the North Sea. Specifically, year was treated as a sample unit, and all scaled residuals within a year were randomly switched in sign. The new residuals were added to the model predictions to fit a new GAM. The operation was repeated 1000 times to estimate mean and confidence intervals for each covariate response. However, the bootstrapped mean effects and confidence intervals were similar to those observed in the models (S2 Appendix). We therefore concluded that accounting for the residual autocorrelation would not alter our conclusions, and kept the original model formulations.

For cod, we also fit separate habitat models that included local capelin density as predictor (*extended habitat models*). These models were used for inference only, not for calculation of overlap.

#### Quantifying overlap

To calculate overlap, the best candidate distribution model for each component was used to predict species density on a 35 x 35 nmi (65 x 65 km) regular grid of the study area with covariate values corresponding to the nearest observation from the central point of the grid cell in year *t*. The grid resolution was the same as the standard inter-station distance of the survey with the coarsest station grid (the ecosystem survey). By using a standard grid, the seasons and years could be compared, despite variation in survey design and execution. Grid cells containing fewer than 5 (autumn) or 8 (winter) observations across the study period, as well as cells falling outside the sampled area in year *t*, were eliminated from the grid. In this way, we only predicted on locations where the models had been given a reasonable amount of data. The overlap *O*_*(x*,*y)*,*t*_ in position (grid cell) *x*,*y* in year *t* was then calculated for each combination of components, using the formula:

$$O_{(x,y),t}=\frac{{\hat{Cap}}_{(x,y),t}}{\text{max}\hat{{Cap}_{t}}}*\frac{{\hat{Cod}}_{(x,y),t}}{\text{max}\hat{{Cod}_{t}}}$$ (4)

Where ${\hat{Cap}}_{(x,y),t}$ and ${\hat{Cod}}_{(x,y),t}$ are the predicted capelin and cod densities in the grid cell, and $\text{max}\hat{{Cap}_{t}}$ and $\text{max}\hat{{Cod}_{t}}$ are the maximum predicted densities in the same year and season. With this formulation, the overlap can range from 0 to 1, where 0 means that one or both species are absent from the grid cell, and 1 means that both species are present in their maximum predicted densities in that year and season. Note that the index is symmetric with respect to species. Thus, our overlap index gave spatially explicit information about how well cod and capelin densities matched in a given year and season. The correlation (Kendall’s rank correlation tau) between the predicted cod and capelin densities across the grid was also calculated for comparison with the spatially explicit overlap formulation. The overlap between all capelin and cod component combinations (autumn: 2, winter :4) were mapped for each year and season.

Finally, the mean overlap across the grid and the extent of the overlap (n grid cells with overlap > 0.001 divided by the total number of grid cells) were calculated for each year, season and cod-capelin component combination to get an overview of the temporal dimension of the overlap, i.e., the between-year variation in how well cod and capelin densities matched.

## Results

### Species distribution models

For all models, the estimated Tweedie power parameters fell within the range 1.4–1.8, indicating that the compound Poisson-gamma distribution was a good fit for our data. The covariates generally contributed significantly to explaining species distributions, except for sun height in the capelin autumn models, and depth in the candidate models of both acoustic and trawl capelin in winter (Table 1). The deviance explained by the best candidate models ranged from 39.6% for capelin trawl data in winter to 74.5% for capelin acoustic data in autumn (Table 1). The relationships between species densities and sun height and survey day are shown in S3 Appendix.

**Table 1:** GAM statistics for all models by season and component (immature cod, mature cod, acoustic capelin, trawl capelin).

Season	Species	Component	Model type	Model terms	Tw-p	ΔAIC	Dev %
**Autumn**	Capelin	*Acoustics*	Habitat	*Base*^a^ + *s*_4_ (*bio*_*t*_) + *s*_5_ (*temp*_(*x*,*y*),*t*_) + *s*_6_ (*s*.*day*_(*x*,*y*),*t*_)	1.452		62.6
			Candidate	*Base*^a^ + *s*_4_ (*x*, *y*) × *bio*_*t*_ + *s*_5_ (*temp*_(*x*,*y*),*t*_) + *s*_6_ (*s*.*day*_(*x*, *y*),*t*_)	1.434	-443.2	69.0
			Candidate	*Base*^a^ + *s*_4_ (*bio*_*t*_) + *s*_5_ (*x*, *y*) × *temp*_(*x*,*y*),*t*_ + *s*_6_ (*s*.*day*_(*x*, *y*),*t*_)	1.45	-206.5	65.3
			Candidate	*Base*^a^ + *s*_4_ (*bio*_*t*_) + *s*_5_ (*temp*_(*x*,*y*),*t*_) + *s*_6_ (*x*, *y*) × *s*.*day*_(*x*,*y*),*t*_	1.432	-470.4	68.8
			Candidate	*Base*^b^ + *s*_4_ (*x*, *y*) × *bio*_*t*_ + *s*_5_ (*x*, *y*) × *temp*(*x*,*y*),*t* + *s*_6_ (*s*.*day*_(*x*,*y*),*t*_)	1.43	-459.4	69.7
			Candidate	*Base*^a^ + *s*_4_ (*x*, *y*) × *bio*_*t*_ + *s*_5_ (*temp*_(*x*,*y*),*t*_) + *s*_6_ (*x*, *y*) × *s*.*day*_(*x*, *y*),*t*_	1.41	-723.5	72.9
			Candidate	*Base*^a^ + *s*_4_ (*bio*_*t*_) + *s*_5_ (*x*, *y*) × *temp*_(*x*,*y*),*t*_ + *s*_6_ (*x*, *y*) × *s*.*day*_(*x*, *y*),*t*_	1.411	-633.5	71.1
			**Candidate**	***Base***^a^ **+ *s***_**4**_ **(*x*, *y*) × *bio***_***t***_ **+ *s***_**5**_ **(*x*, *y*) × *temp***_**(*x*, *y*),*t***_ **+ *s***_**6**_ **(*x*, *y*) ×** (***s***.***day***_(***x***, ***y***),***t***_	**1.399**	**-806.2**	**74.5**
	Cod	*Immature*	Habitat	*Base* + *s*_4_ (*bio*_*t*_) + *s*_5_ (*temp*_(*x*,*y*),*t*_) + *s*_6_ (*s*.*day*_(*x*,*y*),*t*_)	1.62		51.3
			Extended habitat	*Base* + *s*_4_ (*bio*_*t*_) + *s*_5_ (*temp*_(*x*,*y*),*t*_) + *s*_6_ (*s*.*day*_(*x*,*y*),*t*_) + *s*_7_ (*log*_10_ (*capA*_(*x*,*y*),*t*_ + 1))	1.616	-150.8	52.7
			Candidate	*Base* + *s*_4_ (*x*, *y*) × *bio*_*t*_ + *s*_5_ (*temp*_(*x*,*y*),*t*_) + *s*_6_ (*s*.*day*_(*x*,*y*),*t*_)	1.605	-837.9	59.5
			Candidate	*Base* + *s*_4_ (*bio*_*t*_) + *s*_5_ (*x*, *y*) × *temp*_(*x*,*y*),*t*_ + *s*_6_ (*s*.*day*_(*x*,*y*),*t*_)	1.616	-81.5	52.7
			Candidate	*Base* + *s*_4_ (*bio*_*t*_) + *s*_5_ (*temp*_(*x*,*y*),*t*_) + *s*_6_ (*x*, *y*) × (*s*.*day*_(*x*, *y*),*t*_	1.612	-278.3	54.8
			Candidate	*Base* + *s*_4_ (*x*, *y*) × *bio*_*t*_ + *s*_5_ (*x*, *y*) × *temp*_(*x*,*y*),*t*_ + *s*_6_ (*s*.*day*_(*x*, *y*),*t*_)	1.603	-890.5	60.3
			Candidate	*Base* + *s*_4_ (*x*, *y*) × *bio*_*t*_ + *s*_5_ (*temp*_(*x*,*y*),*t*_) + *s*_6_ (*x*, *y*) × *s*.*day*_(*x*, *y*),*t*_	1.597	-969.3	61.4
			Candidate	*Base* + *s*_4_ (*bio*_*t*_) + *s*_5_ (*x*, *y*) × *temp*_(*x*,*y*),*t*_ + *s*_6_ (*x*, *y*) × *s*.*day*_(*x*, *y*),*t*_	1.607	-410.6	56.6
			**Candidate**	***Base* + *s***_**4**_ **(*x*, *y*) × *bio***_***t***_ **+ *s***_**5**_ **(*x*, *y*) × *temp***_**(*x*, *y*),*t***_ **+ *s***_**6**_ **(*x*, *y*) × *s***.***day***_(***x***, ***y***),***t***_	**1.595**	**-1052.0**	**62.5**
		*Mature*	Habitat	*Base* + *s*_4_ (*bio*_*t*_) + *s*_5_ (*temp*_(*x*,*y*),*t*_) + *s*_6_ (*s*.*day*_(*x*, *y*),*t*_)	1.418		47.1
			Extended habitat	*Base* + *s*_4_ (*bio*_*t*_) + *s*_5_ (*temp*_(*x*,*y*),*t*_) + *s*_6_ (*s*.*day*_(*x*,*y*),*t*_) + *s*_7_ (*log*_10_ (*capA*_(*x*,*y*),*t*_ + 1))	1.424	-64.2	48.1
			Candidate	*Base* + *s*_4_ (*x*, *y*) × *bio*_*t*_ + *s*_5_ (*temp*_(*x*,*y*),*t*_) + *s*_6_ (*s*.*day*_(*x*, *y*),*t*_)	1.389	-615.9	55.7
			Candidate	*Base* + *s*_4_ (*bio*_*t*_) + *s*_5_ (*x*, *y*) × *temp*_(*x*,*y*),*t*_ + *s*_6_ (*s*.*day*_(*x*, *y*),*t*_)	1.406	-151.8	50.2
			Candidate	*Base* + *s*_4_ (*bio*_*t*_) + *s*_5_ (*temp*_(*x*,*y*),*t*_) + *s*_6_ (*x*, *y*) × *s*.*day*_(*x*, *y*),*t*_	1.407	-160.1	50.2
			Candidate	*Base* + *s*_4_ (*x*, *y*) × *bio*_*t*_ + *s*_5_ (*x*, *y*) × *temp*_(*x*,*y*),*t*_ + *s*_6_ *s*.*day*_(*x*, *y*),*t*_)^d^	1.379	-694.4	57.3
			Candidate	*Base* + *s*_4_ (*x*, *y*) × *bio*_*t*_ + *s*_5_ (*temp*_(*x*,*y*),*t*_) + *s*_6_ (*x*, *y*) × *s*.*day*_(*x*, *y*),*t*_	1.381	-684.6	57.4
			Candidate	*Base* + *s*_4_ (*bio*_*t*_) + *s*_5_ (*x*, *y*) × *temp*_(*x*,*y*),*t*_ + *s*_6_ (*x*, *y*) × *s*.*day*_(*x*, *y*),*t*_	1.379	-694.4	57.3
			**Candidate**	***Base* + *s***_**4**_ **(*x*, *y*) × *bio***_***t***_ **+ *s***_**5**_ **(*x*, *y*) × *temp***_**(*x*, *y*),*t***_ **+ *s***_**6**_ **(*x*, *y*) × *s***.***day***_(***x***, ***y***),***t***_	**1.372**	**-753.0**	**58.6**
**Winter**	Capelin	*Acoustics*	Habitat	*Base* + *s*_4_ (*bio*_*t*_) + *s*_5_ (*temp*_(*x*,*y*),*t*_) + *s*_6_ (*s*.*day*_(*x*, *y*),*t*_)	1.58		58.9
			Candidate	*Base* + *s*_4_ (*x*, *y*) × *bio*_*t*_ + *s*_5_ (*temp*_(*x*,*y*),*t*_) + *s*_6_ (*s*.*day*_(*x*, *y*),*t*_)	1.576	-79.7	61.6
			Candidate	*Base* + *s*_4_ (*bio*_*t*_) + *s*_5_ (*x*, *y*) × *temp*_(*x*,*y*),*t*_ + *s*_6_ (*s*.*day*_(*x*, *y*),*t*_)	1.555	-212.0	64.3
			Candidate	*Base* + *s*_4_ (*bio*_*t*_) + *s*_5_ (*temp*_(*x*,*y*),*t*_) + *s*_6_ (*x*, *y*) × *s*.*day*_(*x*, *y*),*t*_	1.571	-76.8	61.2
			Candidate	*Base*^c^ + *s*_4_ (*x*, *y*) × *bio*_*t*_ + *s*_5_ (*x*, *y*) × *temp*_(*x*, *y*),*t*_ + *s*_6_ (*s*.*day*_(*x*, *y*),*t*_)^d^	1.557	-249.5	65.6
			Candidate	*Base* + *s*_4_ (*x*, *y*) × *bio*_*t*_ + *s*_5_ (*temp*_(*x*,*y*),*t*_) + *s*_6_ (*x*, *y*) × *s*.*day*_(*x*, *y*),*t*_	1.576	-101.1	62.5
			Candidate	*Base*^c^ + *s*_4_ (*bio*_*t*_) + *s*_5_ (*x*, *y*) × *temp*_(*x*,*y*),*t*_ + *s*_6_ (*x*, *y*) × *s*.*day*_(*x*, *y*),*t*_	1.559	-172.8	64.3
			**Candidate**	***Base***^c^ **+ *s***_**4**_ **(*x*, *y*) × *bio***_***t***_ **+ *s***_**5**_ **(*x*, *y*) × *temp***_**(*x*, *y*),*t***_ **+ *s***_**6**_ **(*x*, *y*) × *s***.***day***_(***x***, ***y***),***t***_	**1.55**	**-295.2**	**67.3**
		*Trawl*	Habitat	*Base* + *s*_4_ (*bio*_*t*_) + *s*_5_ (*temp*_(*x*,*y*),*t*_) + *s*_6_ (*s*.*day*_(*x*, *y*),*t*_)	1.793		31.0
			Candidate	*Base* + *s*_4_ (*x*, *y*) × *bio*_*t*_ + *s*_5_ (*temp*_(*x*,*y*),*t*_) + *s*_6_ (*s*.*day*_(*x*, *y*),*t*_)^d^	1.786	-205.0	35.4
			Candidate	*Base* + *s*_4_ (*bio*_*t*_) + *s*_5_ (*x*, *y*) × *temp*_(*x*,*y*),*t*_ + *s*_6_ (*s*.*day*_(*x*, *y*),*t*_)	1.79	-78.6	33.0
			Candidate	*Base* + *s*_4_ (*bio*_*t*_) + *s*_5_ (*temp*_(*x*,*y*),*t*_)+ *s*_6_ (*x*, *y*) × *s*.*day*_(*x*, *y*),*t*_	1.786	-236.4	35.7
			Candidate	*Base*^c^ + *s*_4_ (*x*, *y*) × *bio*_*t*_ + *s*_5_ (*x*, *y*) × *temp*_(*x*,*y*),*t*_ + *s*_6_ (*s*.*day*_(*x*, *y*),*t*_)^d^	1.784	-250.7	36.5
			Candidate	*Base*^c^ + *s*_4_ (*x*, *y*) × *bio*_*t*_ + *s*_5_ (*temp*_(*x*,*y*),*t*_)^d^ + *s*_6_ (*x*, *y*) × *s*.*day*_(*x*, *y*),*t*_	1.782	-311.2	37.5
			Candidate	*Base*^c^ + *s*_4_ (*bio*_*t*_) + *s*_5_ (*x*, *y*) × *temp*_(*x*,*y*),*t*_ + *s*_6_ (*x*, *y*) × *s*.*day*_(*x*, *y*),*t*_	1.784	-248.8	36.3
			**Candidate**	***Base***^c^ **+ *s***_**4**_ **(*x*, *y*) × *bio***_***t***_ **+ *s***_**5**_ **(*x*, *y*) × *temp***_**(*x*, *y*),*t***_ **+ *s***_**6**_ **(*x*, *y*) × *s***.***day***_(***x***, ***y***),***t***_	**1.779**	**-400.6**	**39.6**
	Cod	*Immature*	Habitat	*Base* + *s*_4_ (*bio*_*t*_) + *s*_5_ (*temp*_(*x*,*y*),*t*_) + *s*_6_ (*s*.*day*_(*x*, *y*),*t*_)	1.658		50.4
			Extended habitat	*Base* + *s*_4_ (*bio*_*t*_) + *s*_5_ (*temp*_(*x*,*y*),*t*_) + *s*_6_ (*s*.*day*_(*x*,*y*),*t*_) + *s*_7_ (*log*_10_ (*capA*_(*x*,*y*),*t*_ + 1))	1.657	+10.0	50.6
			Extended habitat	*Base* + *s*_4_ (*bio*_*t*_) + *s*_5_ (*temp*_(*x*,*y*),*t*_) + *s*_6_ (*s*.*day*_(*x*,*y*),*t*_) + *s*_7_ (*log*_10_ (*capT*_(*x*,*y*),*t*_ + 1))	1.655	-48.7	51.2
			Candidate	*Base* + *s*_4_ (*x*, *y*) × *bio*_*t*_ + *s*_5_ (*temp*_(*x*,*y*),*t*_) + *s*_6_ (*s*.*day*_(*x*, *y*),*t*_)^d^	1.661	-204.1	53.5
			Candidate	*Base* + *s*_4_ (*bio*_*t*_) + *s*_5_ (*x*, *y*) × *temp*_(*x*,*y*),*t*_ + *s*_6_ (*s*.*day*_(*x*, *y*),*t*_)	1.65	-165.6	53.0
			Candidate	*Base* + *s*_4_ (*bio*_*t*_) + *s*_5_ (*temp*_(*x*,*y*),*t*_) + *s*_6_ (*x*, *y*) × *s*.*day*_(*x*, *y*),*t*_	1.654	-54.6	51.9
			Candidate	*Base* + *s*_4_ (*x*, *y*) × *bio*_*t*_ + *s*_5_ (*x*, *y*) × *temp*_(*x*,*y*),*t*_ + *s*_6_ (*s*.*day*_(*x*, *y*),*t*_)^d^	1.661	-201.1	54.0
			Candidate	*Base* + *s*_4_ (*x*, *y*) × *bio*_*t*_ + *s*_5_ (*temp*_(*x*,*y*),*t*_) + *s*_6_ (*x*, *y*) × *s*.*day*_(*x*, *y*),*t*_	1.664	-81.6	52.8
			Candidate	*Base* + *s*_4_ (*bio*_*t*_) + *s*_5_ (*x*, *y*) × *temp*_(*x*,*y*),*t*_ + *s*_6_ (*x*, *y*) × (*s*.*day*_(*x*, *y*),*t*_	1.657	-26.1	52.1
			**Candidate**	***Base* + *s***_**4**_ **(*x*, *y*) × *bio***_***t***_ **+ *s***_**5**_ **(*x*, *y*) × *temp***_**(*x*, *y*),*t***_ **+ *s***_**6**_ **(*x*, *y*) × *s***.***day***_(***x***, ***y***),***t***_	**1.657**	**-284.5**	**55.5**
		*Mature*	Habitat	*Base* + *s*_4_ (*bio*_*t*_) + *s*_5_ (*temp*_(*x*,*y*),*t*_) + *s*_6_ (*s*.*day*_(*x*, *y*),*t*_)	1.533		54.2
			Extended habitat	*Base* + *s*_4_ (*bio*_*t*_) + *s*_5_ (*temp*_(*x*,*y*),*t*_) + *s*_6_ (*s*.*day*_(*x*,*y*),*t*_) + *s*_7_ (*log*_10_ (*capA*_(*x*,*y*),*t*_ + 1))	1.537	+99.3	53.1
			Extended habitat	*Base* + *s*_4_ (*bio*_*t*_) + *s*_5_ (*temp*_(*x*,*y*),*t*_) + *s*_6_ (*s*.*day*_(*x*,*y*),*t*_) + *s*_7_ (*log*_10_ (*capT*_(*x*,*y*),*t*_ + 1))	1.537	+102.8	53.0
			Candidate	*Base* + *s*_4_ (*x*, *y*) × *bio*_*t*_ + *s*_5_ (*temp*_(*x*,*y*),*t*_) + *s*_6_ (*s*.*day*_(*x*, *y*),*t*_)	1.541	-114.0	55.3
			Candidate	*Base* + *s*_4_ (*bio*_*t*_) + *s*_5_ (*x*, *y*) × *temp*_(*x*,*y*),*t*_ + *s*_6_ (*s*.*day*_(*x*, *y*),*t*_)	1.535	+46.2	54.0
			Candidate	*Base* + *s*_4_ (*bio*_*t*_) + *s*_5_ (*temp*_(*x*,*y*),*t*_) + *s*_6_ (*x*, *y*) × *s*.*day*_(*x*, *y*),*t*_	1.534	+50.7	54.1
			Candidate	*Base* + *s*_4_ (*x*, *y*) × *bio*_*t*_ + *s*_5_ (*temp*_(*x*,*y*),*t*_) + *s*_6_ (*x*, *y*) × *s*.*day*_(*x*, *y*),*t*_	1.539	-78.8	55.6
			Candidate	*Base* + *s*_4_ (*x*, *y*) × *bio*_*t*_ + *s*_5_ (*x*, *y*) × *temp*_(*x*,*y*),*t*_ + *s*_6_ (*s*.*day*_(*x*, *y*),*t*_)	1.538	-101.0	55.7
			Candidate	*Base* + *s*_4_ (*bio*_*t*_) + *s*_5_ (*x*, *y*) × *temp*_(*x*,*y*),*t*_ + *s*_6_ (*x*, *y*) × *s*.*day*_(*x*, *y*),*t*_	1.538	+145.3	53.0
			**Candidate**	***Base* + *s***_**4**_ **(*x*, *y*) × *bio***_***t***_ **+ *s***_**5**_ **(*x*, *y*) × *temp***_**(*x*, *y*),*t***_ **+ *s***_**6**_ **(*x*, *y*) ×** *s*.*day*_(***x***, ***y***),***t***_	**1.533**	**-195.8**	**57.4**

The terms for spatial position, s_1_(*x*,*y*), sun height, s_2_(*sun*_*(x*,*y)*,*t*_), and depth, s_3_(*depth*_*(x*,*y)*_), were included in all models and are denoted “Base” in the table. Tw-p is the estimated Tweedie power parameter. Deviance explained (Dev %) is presented for the final model after removal of non-significant (n.s., p > 0.05) terms, and ΔAIC is the change in AIC relative to the habitat model for each component. The extended habitat models included local capelin density as predictor; here capA represents capelin sampled with acoustics and capT represents capelin caught in the bottom trawl. The chosen candidate model for each component is indicated in bold font.

^a^Sunheight n.s.

^b^Sunheight and depth n.s.

^c^Depth n.s

^d^n.s. term

#### Factors affecting species distributions in autumn

**Fig 3:**
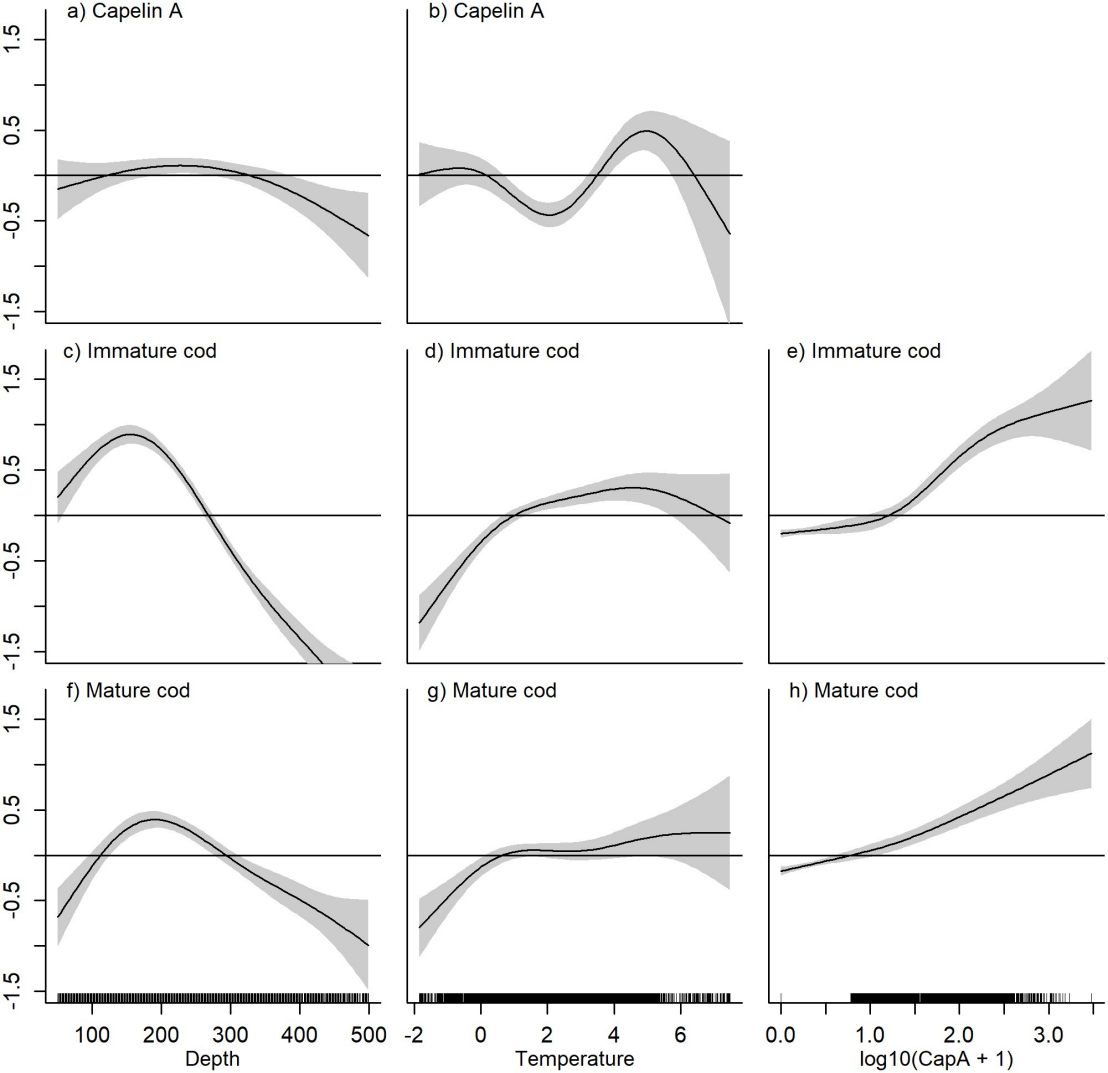
Autumn GAM smooth functions from the habitat models. Non-linear regression between local densities of A-B) capelin, C-D) immature cod, and F-G) mature cod and the density-independent covariates depth (m) and temperature (°C). The effect of local capelin density (log_10_[NASC+1]) from the extended habitat models on E) immature cod density and H) mature cod density is also shown. The plot shows the (centered) log local species density as a function of each covariate when accounting for the other covariate effects. The horizontal line at y = 0 represents a neutral contribution of the covariate to the response. The grey bands represent ± 2 standard errors around the smooth estimate.

The estimated relationships between local densities and depth and temperature from the habitat models in autumn (Table 1) are shown in Fig 3. Capelin did not associate strongly with bottom depth, but occurred in lower than average densities in the deepest areas (Fig 3A). The relationship between temperature and capelin density was bimodal: higher capelin densities were found in sub-zero waters, and in temperatures of around 5°C (Fig 3B). However, the confidence intervals for depth and temperature were relatively wide, and the bootstrapped confidence intervals resulted in non-significant p-values (S2 Appendix).

**Fig 4:**
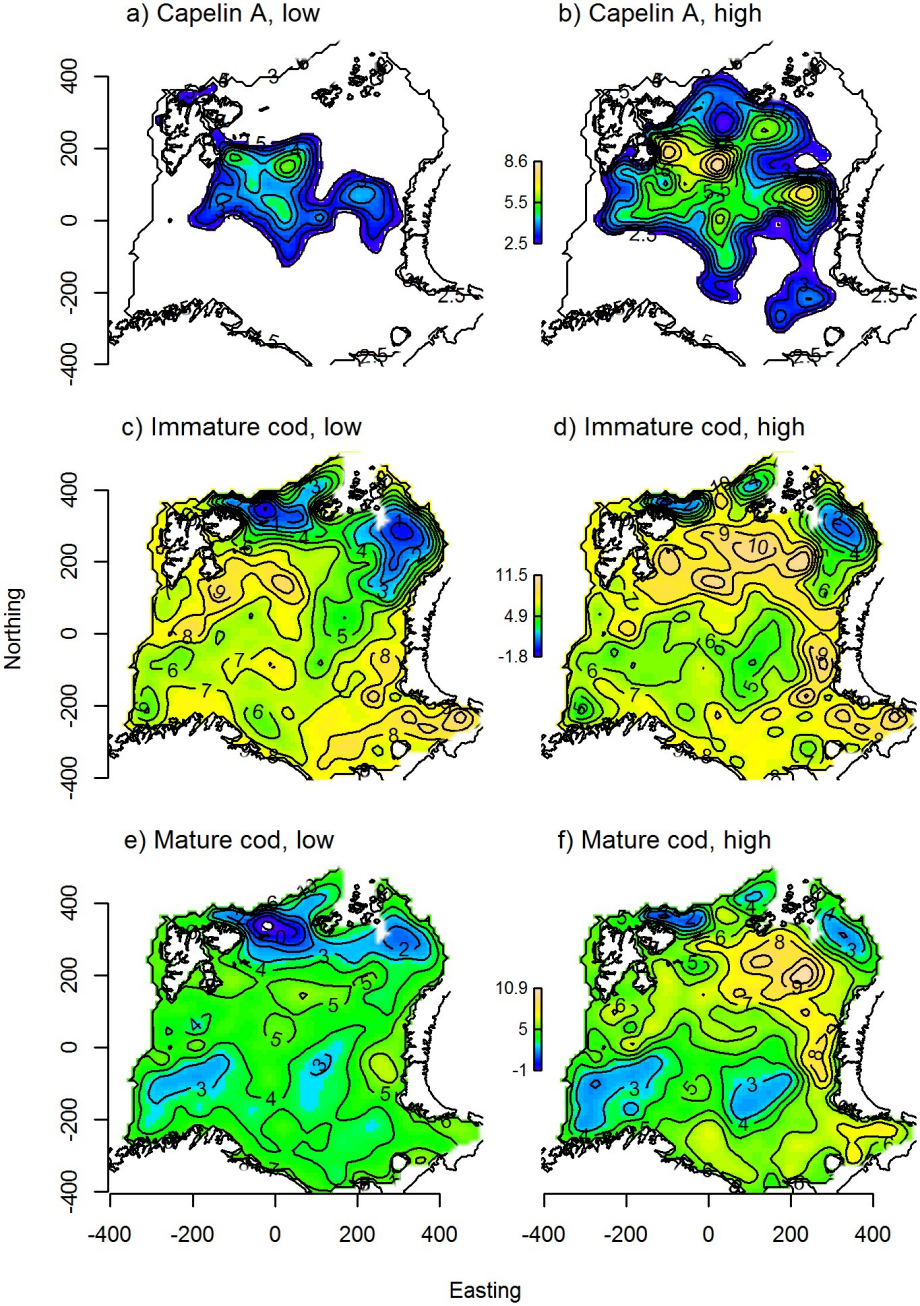
Predicted autumn distributions. Autumn distributions of A-B) capelin, C-D) immature cod, and E-F) mature cod, as predicted from the best candidate model for each component (Table 1). The different columns show the partial effects of stock biomass when the other model predictors were set to their across-year mean values at each location; the left column shows species distributions at low stock biomass (capelin: 0.628, cod: 1.63 million tonnes, measured in 2004), and the right at high stock biomass (capelin: 3.96, cod: 4.38 million tonnes, measured in 2013). The contour lines indicate local species density on the log-link scale, and the colours range from blue at low density to yellow at high density.

**Fig 5:**
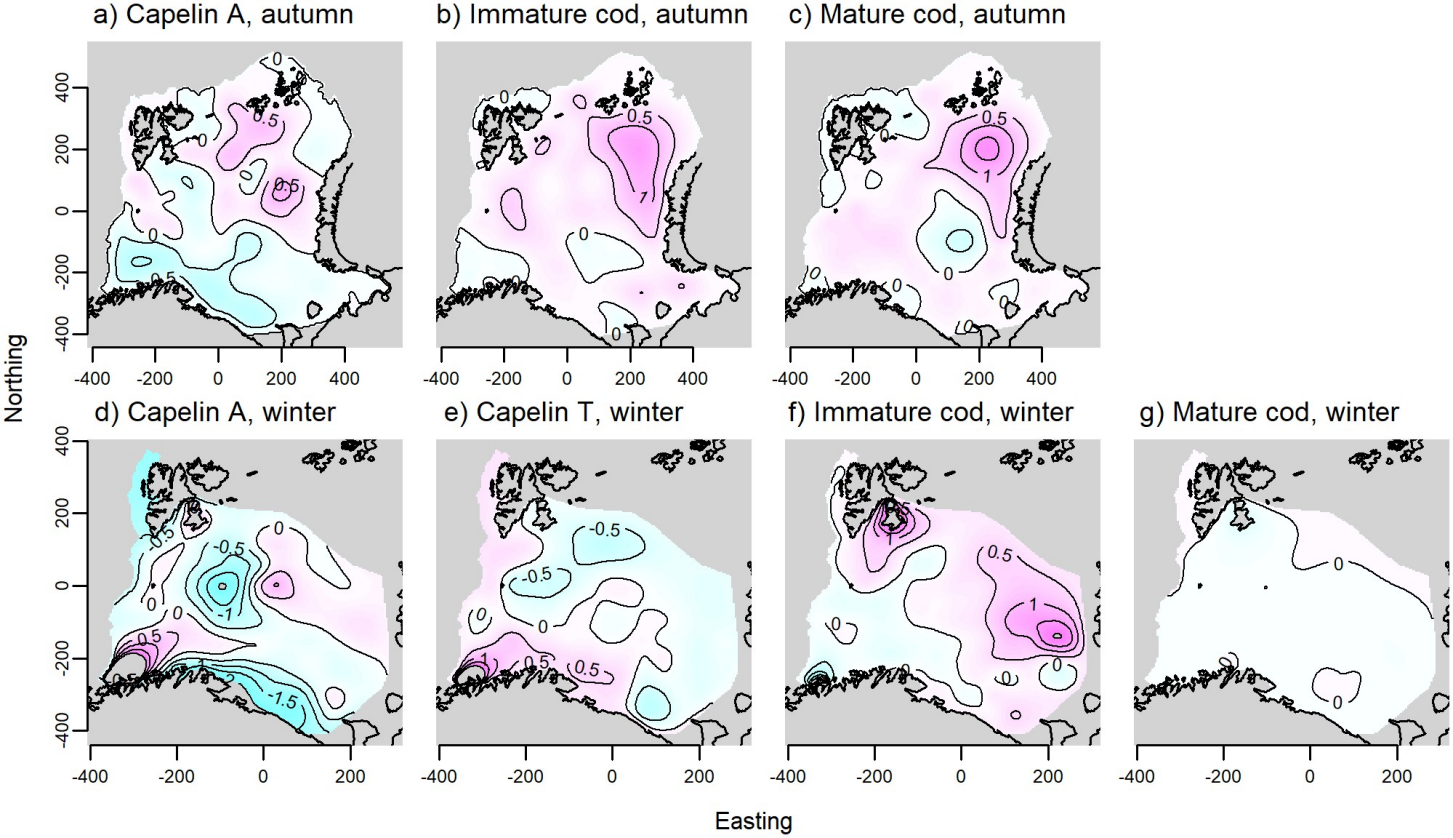
Spatially variant effect of temperature on local cod and capelin densities. The contour lines show how the slope of the linear regression between local species density and mean pelagic temperature (capelin) or bottom temperature (cod) from the best candidate models vary in space for A) acoustically estimated capelin in autumn, B) immature cod in autumn, C) mature cod in autumn, D) acoustically estimated capelin in winter, E) trawl-caught capelin in winter, F) immature cod in winter, and G) mature cod in winter. Blue colours indicate negative slopes, and pink colours indicate positive slopes.

Capelin was mainly restricted to the central-northern parts of the Barents Sea, with a core distribution area east of Svalbard (Fig 4A). For capelin, an increase in stock biomass lead to an expansion of the core distribution area towards the north and south, as well as density increases in the core area and farther east (Fig 4A and 4B). This effect was significant across all models. Including a spatially variant effect of temperature further improved model fit, as increased temperature in the north-eastern area was associated with higher local capelin densities (Fig 5A). The final model for capelin in autumn explained 74.5% of the deviance and included, in addition to the effects described above, a locally linear effect of survey day (Table 1, S3 Appendix).

Immature cod occurred in areas with slightly shallower bottom depths compared to mature cod (Fig 3C and 3F). Peak densities of both components fell within the range 150–200 m. Less than average cod densities were found in temperatures below 1°C, but above that any effect of temperature on mature cod was generally weak and variable (Fig 3G), while immature cod associated more strongly with water masses of intermediate temperature (2–5°C, Fig 3D). Including local capelin density as a predictor of local cod density, we found a positive association, though the effect was more variable for immature than mature cod at high capelin densities (Fig 3E and 3H). Including capelin gave a modest improvement in model fit compared to the basic habitat models (1–2% increase in deviance explained, Table 1). Both immature and mature cod were found throughout the study area, with density maxima both in central-northern and south-eastern Barents Sea (Fig 4C and 4E). The distributions of mature and immature cod were similar, but the highest mature cod densities occurred slightly east of the immature cod density maximum in the north. The spatially variant effect of cod stock biomass on cod density was significant across model formulations for both immature and mature cod. As stock biomass increased, the main density increases for both components occurred in the north/north-eastern part of the study area (Fig 4C–4F). Including a spatially variant effect of bottom temperature further improved model fit (Table 1). Here, an increase in density of both components coincided with increasing bottom temperatures in the north-easternmost corner of the study area (Fig 5B and 5C). Finally, spatially variant effects of survey day were also retained in the final models for cod in autumn, which explained 62.5% and 58.6% of the deviance for immature and mature cod, respectively (Table 1, S3 Appendix).

For all autumn models, the effect size of stock biomass was larger than that of temperature as judged by the difference in AIC between the habitat models and the candidate models with the respective spatially variant term (Table 1).

#### Factors affecting species distributions in winter

**Fig 6:**
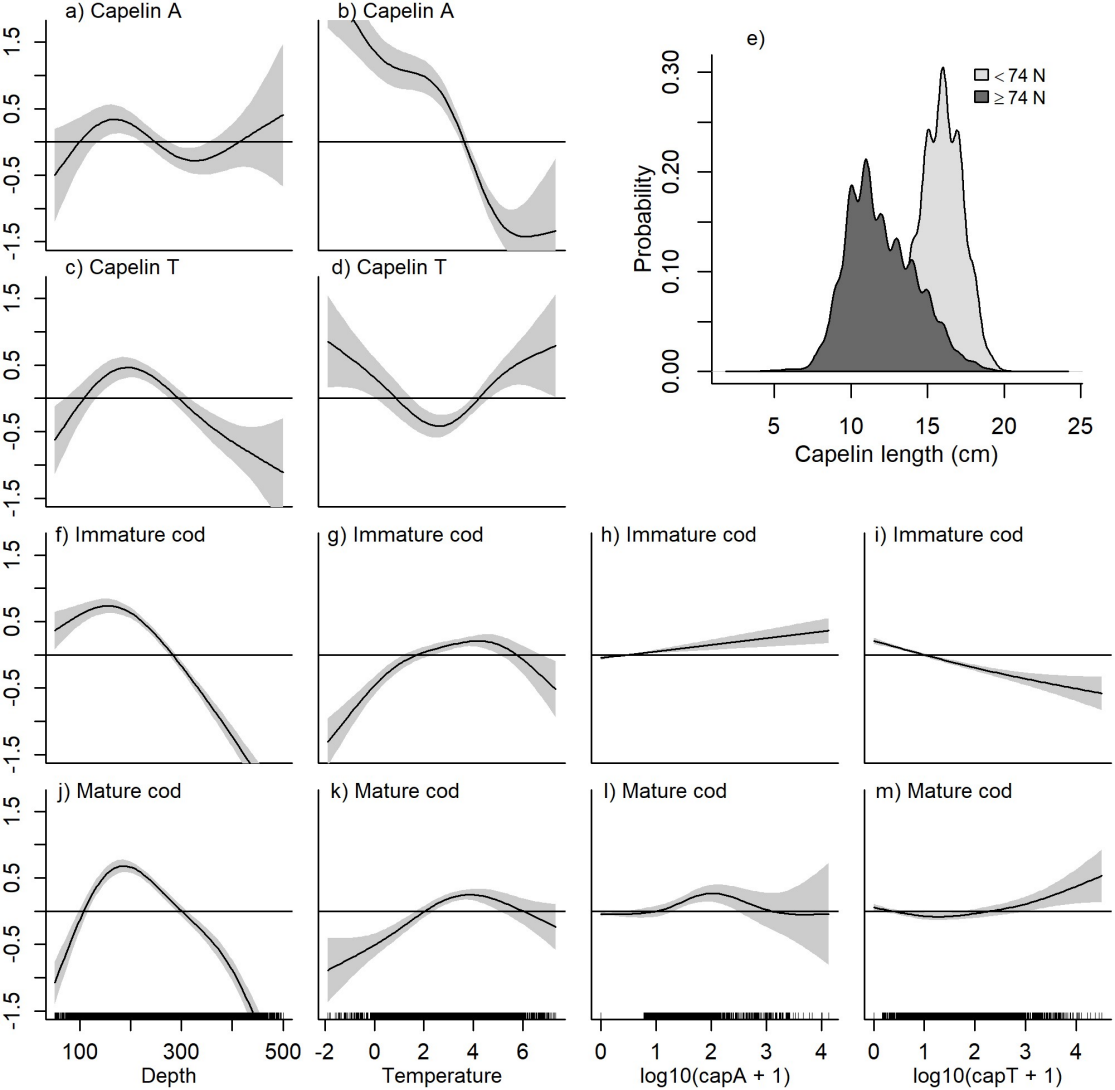
Winter GAM smooth functions from the habitat models. Non-linear regression between local densities of A-B) capelin sampled acoustically, C-D) capelin sampled with demersal trawl, F-G) immature cod, J-K) mature cod, and depth (m) and temperature (°C). The effect of local acoustic (log_10_[NASC+1]) and trawl capelin (log_10_[ind x nmi^-2^+1]) densities from the extended habitat models are shown for H-I) immature cod and L-M) mature cod. The plot shows the (centered) log local species density as a function of the covariate when accounting for the other covariate effects. The horizontal line at 0 corresponds to a neutral contribution of the covariate to the response. The grey bands illustrate ± 2 standard errors around the smooth estimate. Panel E) shows probability density distributions of capelin length in demersal trawl hauls south and north of 74°. The distributions were calculated from the catch numbers of capelin in each 1 cm-length group using R base function “density” with default settings. Capelin matures at approximately 14 cm [23].

The estimated relationships between local species densities and depth and temperature from the habitat models in winter (Table 1) are shown in Fig 6. Higher than average densities of acoustic capelin were found in shallow areas and in the deepest areas, though variability in the response was high at large depths (Fig 6A, see also S2 Appendix; depth was non-significant after the wild bootstrap). Trawl capelin occupied the shallow part of the depth range (Fig 6C), but note that in the best candidate models, depth was non-significant for both capelin components (Table 1). The acoustic capelin was strongly and positively associated with the coldest waters (< 2°C, Fig 6B), reflecting the northern distribution, while the highest trawl catches of capelin coincided with the lowest *and* highest temperatures, but not those in between (Fig 6D). However, the number of observations at these temperature extremes were relatively few, and there was high variability in the response.

**Fig 7:**
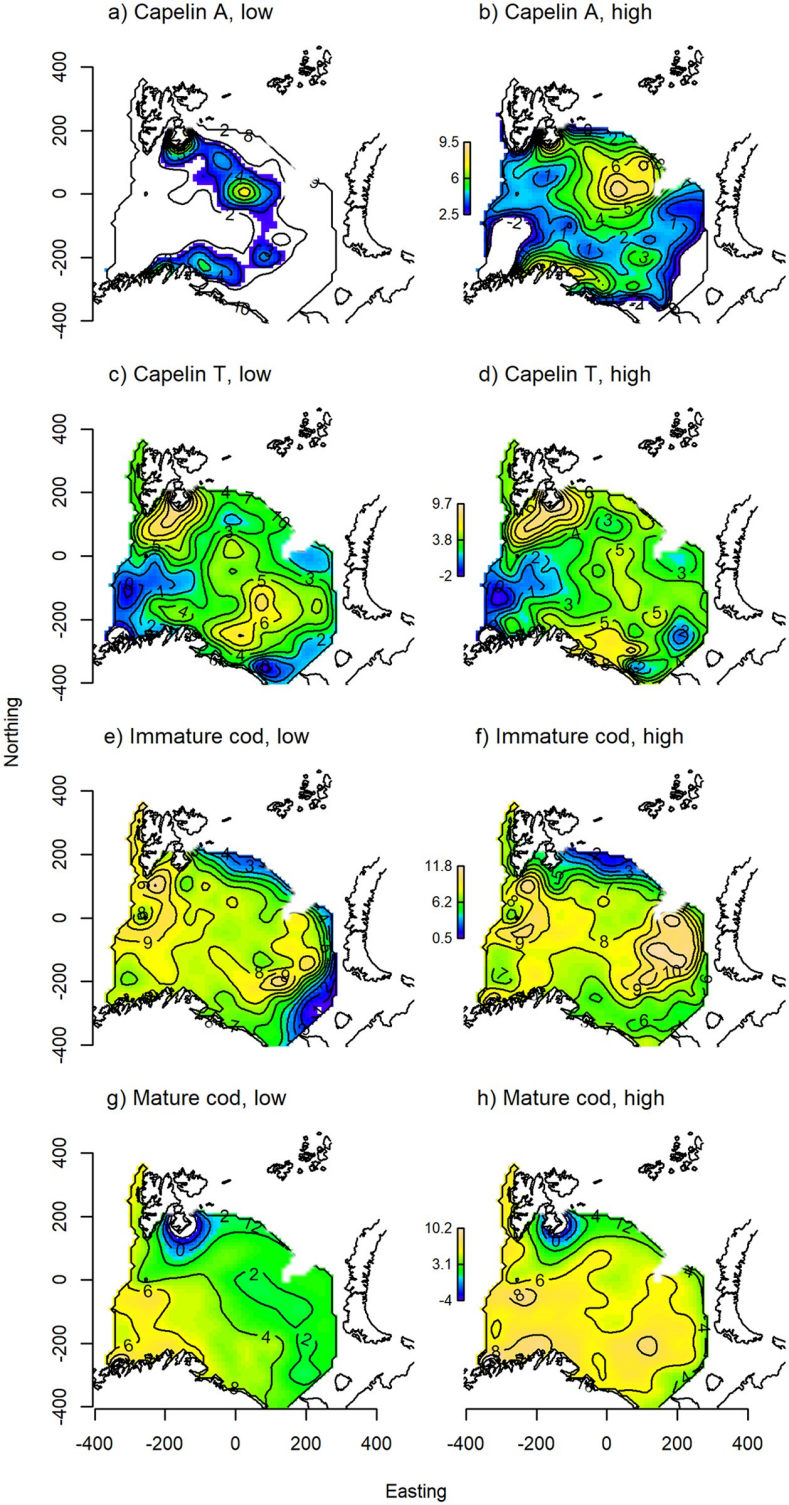
Predicted winter distributions. Winter distributions of A-B) acoustically estimated capelin, C-D) trawl-caught capelin, E-F) immature cod, and G-H) mature cod from the best candidate model for each component (Table 1). The different columns show the partial effects of stock biomass when the other model predictors were set to their across-year mean values in each location; the left column shows species distributions at low stock biomass (capelin: 0.628, cod: 1.63 million tonnes, measured in 2004), and the right at high stock biomass (capelin: 3.96, cod: 4.38 million tonnes, measured in 2013). The contour lines indicate local species density on the log-link scale, and the colours range from blue at low density to yellow at high density.

Partly different geographic distributions were evident from the two sampling methods; the main concentrations of capelin sampled acoustically were found in the central Barents Sea (Fig 7A and 7B), while high densities were caught in the demersal trawl around Svalbard, but also in an area extending across the central areas down to the Norwegian/Russian coasts (Fig 7C and 7D). Smaller individuals dominated the demersal trawl catch in the north, while larger individuals dominated in hauls from the south (Fig 6E). Comparatively lower densities of capelin were measured acoustically along the coast (Fig 7A).

The best models for capelin (both acoustics and trawl) in winter included spatially variant effects of temperature (Table 1). For acoustic capelin, there were negative effects of temperature in the central Barents Sea and along the eastern Norwegian/Russian coasts (Fig 5D), while for trawl capelin, local density decreased with temperature in the north, and increased with temperature in the south (Fig 5E). The effect size of temperature was larger than that of biomass for acoustic capelin, while the biomass effect was larger for trawl capelin (Table 1). The final models explained 67.3% and 39.6% of the deviance for acoustic and trawl capelin, respectively, and also included spatially variant effects of survey day (S3 Appendix) and capelin biomass (Fig 7A–7D).

In winter, immature and mature cod were associated with similar depths as in autumn, that is between 150–200 m (Fig 6F and 6J), while they occupied a narrower and warmer range of temperatures in winter (approx. 2–6°C Fig 6G and 6K). The overall association between acoustic capelin and both immature and mature cod was weak in winter (Fig 6H and 6L). Immature cod had a negative association with trawl capelin (Fig 6I), while mature cod was positively associated with the highest trawl capelin densities (Fig 6M). However, including capelin (either trawl or acoustics) as a predictor contributed little to improving model fits, or even reduced the explained deviance (Table 1).

Cod was found throughout the study area, with density peaks of immature cod in the western- and easternmost areas (Fig 7E) while mature cod occurred in higher densities closer to the Norwegian coast (Fig 7G). Stock biomass was important for explaining variation in the local density of both cod components; the areas of high immature cod density in the east expanded as stock biomass increased (Fig 7E and 7F), while mature cod density increased with stock biomass throughout most of the surveyed area (Fig 7G and 7H). Increased local temperature was associated with an increase in immature cod density in the north and east, and a weak decrease in density in the south-west (Fig 5F). Temperature had a small positive effect on mature cod density in the north (Fig 5G). The effect size of stock biomass was larger than that of temperature for both components (Table 1). The final models for immature and mature cod in winter also included spatially variant effects of survey day (S3 Appendix), and explained 55.5% and 57.4% of the deviance, respectively (Table 1).

### Cod-capelin overlap

**Fig 8:**
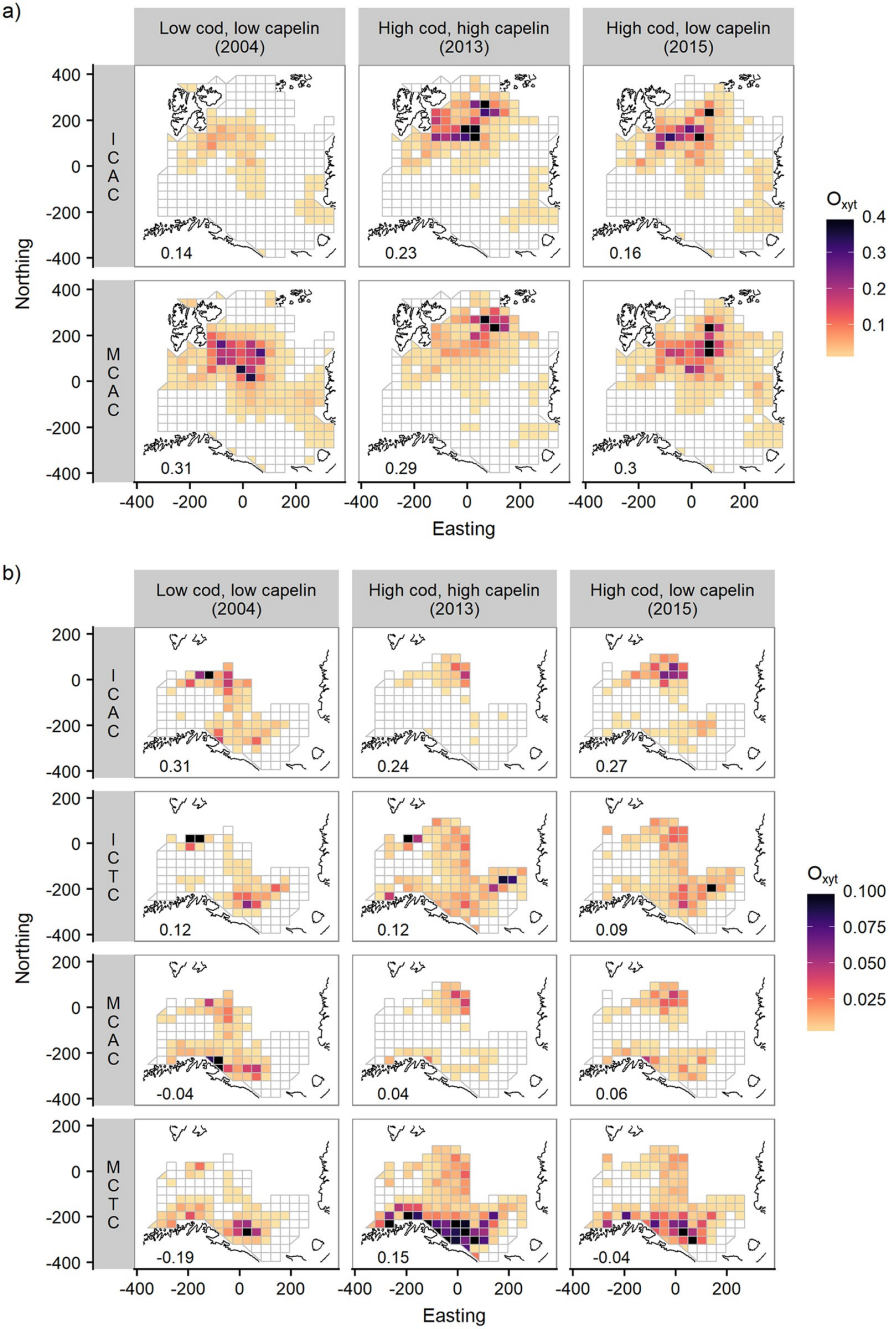
Cod-capelin overlap by season. Overlap (*O*_*(x*,*y)t*_) by component at contrasting cod and capelin biomass for A) autumn and B) winter, calculated on model predictions from the best candidate models. ICAC = immature cod and acoustic capelin, ICTC = immature cod and trawl capelin, MCAC = mature cod and acoustic capelin, MCTC = mature cod and trawl capelin. Overlap values > 0.4 (n = 11) in autumn and > 0.1 (n = 20) in winter were set to black colour to enable good visualisation of the variation in the main overlap range. Note the different ranges of the colour scales in the two seasons. The values in the bottom left corners of each panel is the correlation coefficient (Kendall’s tau) between the predicted cod and capelin densities across the grid.

**Fig 9:**
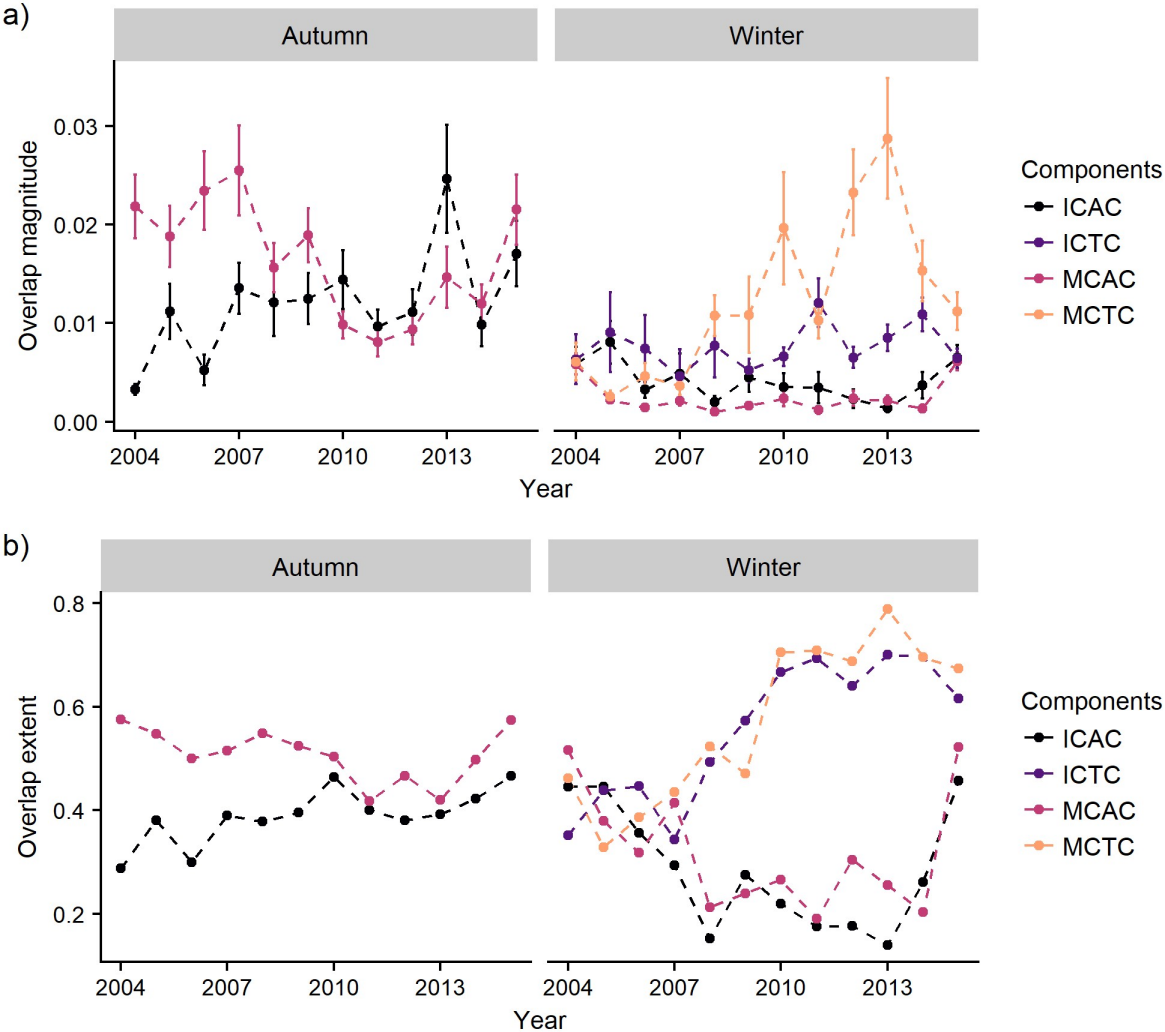
Temporal trends in the overlap. Mean overlap across the grid (magnitude, upper panels) and overlap extent (number of grid cells with overlap > 0.001 divided by the total number of grid cells, lower panels), by year, season, and component pair. ICAC = immature cod and acoustic capelin, ICTC = immature cod and trawl capelin, MCAC = mature cod and acoustic capelin, MCTC = mature cod and trawl capelin. The error bars show 95% confidence intervals of the mean. The sharp dip in the autumn ICAC overlap in 2014 is likely due to incomplete coverage of the immature cod component [27].

Maps of overlap by year and season for all cod-capelin component combinations can be found in S4 Appendix. Maps of overlap in years with contrasting capelin and cod stock biomasses are shown in Fig 8 for autumn and winter, respectively. The mean annual overlap and overlap extent are shown in Fig 9.

#### Cod-capelin overlap in autumn

Capelin was distributed in a comparatively smaller area than cod, mainly restricted to the central-northern parts of the Barents Sea (Fig 4). The main overlap area between cod and capelin coincided with the main distribution area of capelin in all years (Fig 8A, S3 Appendix). The mean overlap was higher between mature cod and capelin compared to the immature cod and capelin overlap in the beginning of the time series, but became similar towards the end as the overlap between immature cod and capelin increased (Fig 9, upper left panel). The overlap between immature cod and capelin was low when both stocks were at a relatively low level (2004), high when both stocks were at a high level (2013) and remained high as the cod stock remained at a high level and the capelin stock had collapsed (2015, Fig 8A, upper panel). There were less clear temporal trends in the overlap between mature cod and capelin (Fig 8A, lower panel). The overlap extent (number of grid cells with overlap > 0.001) between immature cod and capelin showed a positive trend across the study period, while the extent was more variable for the mature cod-capelin overlap (Fig 9, lower left panel).

#### Cod-capelin overlap in winter

Cod had a wider distribution than capelin also in winter (Fig 7). Immature cod overlapped with acoustic capelin mainly near the northern limit of the area covered by the winter survey, except in the first years when they also overlapped farther south (Fig 8B, upper panels, S3 Appendix). There was also a region of overlap between immature cod and trawl capelin in the central-eastern part of the surveyed area and along the coast of Norway and Russia (Fig 8B, second row panels). Mature cod had a more southerly distribution than immature cod and this was reflected in the overlap with capelin. The highest overlap was along the Russian and Norwegian coasts; this was particularly pronounced for overlap with trawl capelin (Fig 8B, lower panels). There was also some overlap with acoustic capelin in the south and north (Fig 8B, third row panels). The highest mean overlap in winter was between mature cod and trawl capelin, and this overlap increased over time (Fig 9, upper right panel). The overlap extent was highly variable in winter for all component pairs, but increased over time for both the immature and mature cod-trawl capelin components (Fig 9, lower right panel).

## Discussion

This is the first study to explicitly estimate overlap between cod and capelin in the Barents Sea. While overlap does not on its own imply consumption, the spatial pattern of overlap tells us where cod and capelin are more likely to interact as predator and prey. We found that overlap varied with season; the main overlap areas were east of Svalbard in autumn, and south of Svalbard and along the Norwegian/Russian coasts in winter. In autumn, the overlap area shifted towards the north-east during the study period. This could be attributed to increased cod stock biomass, and to a lesser extent, increased capelin stock biomass and increased temperature in this area. The autumn overlap remained high after the capelin stock collapse at the end of our study period. The spatial pattern of overlap in winter reflected the disjunct distribution of capelin when matures migrate towards the southern coasts of the Barents Sea to spawn and immatures remain closer to the autumn distribution area (discussed below).

### Methodological considerations

The autumn survey has been designed to collect synoptic data on several trophic levels [29], while the winter survey has demersal fish as primary target. Therefore, factors related to winter survey methodology may influence the capelin part of the spatial analysis. In winter, mature capelin may migrate in the acoustic blind zones close to the bottom or close to the surface [9]. We therefore complemented the acoustic data with demersal trawl data, which include individuals in the acoustic blind zone at the bottom but not at the surface. The two data sources could not be combined; target trawl hauls for capelin are too few to reliably convert acoustic backscatter to biomass of immature and mature capelin at the resolution we used to study overlap. However, on a broad scale, the length distribution in demersal trawl samples is consistent with the generally acknowledged distribution of capelin in winter, and we used this to aid interpretation of the winter results (see below). The limitations of the winter survey data on capelin should be kept in mind when interpreting the results (but see [49, 50]).

In the present study, a main aim was to investigate spatial match between cod and capelin densities. For this purpose, the overlap index was defined such that high values of overlap at any given location resulted from high density of both species. Moreover, in order to express seasonal and inter-annual variation in overlap at a comparable scale, we considered overlap relative to densities within—not across—each year and season (see also [51] for a similar scaling approach). Having an index with the above-mentioned characteristics allowed us to assess spatial changes over time. Various indices of cod-capelin overlap have been applied in previous studies in other areas, with characteristics reflecting the objectives of the investigations. Ciannelli and Bailey [43] applied the product of species densities at a given location. Rose and O'Driscoll [52] applied the number of capelin available to cod within a specific radius. For future studies, it could be valuable to complement our index with, e.g., the potential contact index [53] to examine the number of capelin available to cod within a radius relevant to cod foraging. We may then be able to determine how the magnitude of our overlap index relates to the strength of potential predator-prey interaction.

### Seasonal and temporal trends in the overlap

We found seasonal differences both in where and how strongly cod and capelin overlapped. The generally lower overlap in winter could partly be due to under-sampling of capelin in winter, particularly with acoustics (see above). However, differences in overlap between seasons are expected due to the seasonal variation in behaviour of both species. In autumn, feeding has high priority for cod and capelin, and both species remain in the feeding areas throughout the survey. We found that the autumn overlap was concentrated to the east of Svalbard for both immature and mature cod, and the overlap area moved towards the northeast during the study. The capelin stock was in a state of collapse during the last year of our study period, but the estimated consumption of capelin by cod remained high [27]. This is consistent with our result on autumn overlap which remained high in the year of collapse.

In contrast, in winter, immature and mature individuals of both species differ in their spatial preferences. Immature capelin overwinter in the northernmost ice-free areas of the Barents Sea, whereas mature capelin separate from the rest of the stock to start their spawning migration to the southern coasts [9]. Immature cod following migrating mature capelin to the coast of northern Norway have sustained a traditional spring fishery on cod for centuries [54]. Mature cod spawn along the northwest coast of Norway somewhat later than capelin, but they start migrating towards the spawning grounds around the time of the winter survey [25]. Mature cod feed when they are still inside the Barents Sea, while feeding is reduced on the spawning grounds [55]. We found that the overlap area with acoustic capelin was disjunct, with one overlap area southeast of Svalbard and one along the coast. Based on the length distribution in trawl samples (Fig 6E), we interpret the first overlap area as immature capelin (<14 cm, [23]) overlapping with cod. The cod here were immatures that had not followed mature capelin to their spawning sites, and mature cod that either had not started spawning migration or skipped spawning [56]. The second overlap area along the coast was between both immature and mature cod and mature capelin. For trawl capelin (and acoustic capelin in the first year, Fig 8B), the two areas were connected through the central parts of the surveyed area, and it is likely that the overlap here was with migrating capelin individuals (c.f. Fig 8 in [9]). Therefore, while capelin appears to be relatively more important as prey during winter (comprising 30–60% of the diet in winter, and 15–30% in autumn, study years 2004–2013, [15]), the overlap was more spatially and temporally variable than in autumn.

### Constraints on the overlap

Prey availability to predators may be constrained by physiological adaptations to factors such as depth and/or temperature that differ from those of the prey (e.g., [57]). The prey can benefit from these constraints and find refuges, resulting in reduced predator-prey overlap (e.g., [3]). In the present study, we tested if the occupied habitat differed between cod and capelin by including temperature and depth in the distribution models. Differences in habitat could imply spatial refuges for capelin from cod. In autumn, no indication of refugia with respect to temperature for capelin was found, as cod and capelin occupied similar temperature ranges (Fig 3). The result contrasts with findings from other cod-capelin systems. In the Bering Sea, with co-occurrence of the Pacific cod (*Gadus macrocephalus*) and capelin, the cod-capelin link is much weaker than in the Atlantic ecosystems. This weak link is the result of a cold pool that in some years keeps cod confined to the warmer waters on the southern shelf while capelin finds a refuge in the north [43]. In the Newfoundland-Labrador ecosystem, cod were spatially constrained to intermediate temperatures while capelin had a refuge in both the coldest and warmest waters [3]. Off Iceland, observed reduction in cod-capelin overlap during autumn in the early 2000s was related to increased inflow of warm Atlantic water triggering capelin to migrate farther off the shelf into deeper waters where cod did not follow [58, 59]. We found depth-related constraints for cod, but not so for capelin. A refugium in deep waters for capelin is thus possible, potentially due to costs of maintaining neutral buoyancy for cod in the deep ([60], and references therein).

Our results from the winter regarding overlapping habitats and spatial refuges were less clear than the results from autumn. The across-space correlations between cod and capelin densities were weak or negative in some years, reflecting the complex spatial distribution of the capelin stock (Figs 6I and 8, and S4 Appendix, values in bottom left corner of each panel). Parts of the capelin stock occurred in the coldest waters while cod appeared to avoid these water masses, providing capelin with a refugium (Fig 6). The density of trawl capelin increased along the coast in the south when local temperature increased, while the distribution of immature cod shifted north (Fig 5E and 5F). These reverse patterns suggest that increased temperature reduced the overlap in the north. The higher capelin densities in the south with higher water temperatures might be caused by earlier spawning migration in warm years [61].

Generally, the match between cod and capelin densities was low; the overlap never reached maximum value (across all years, two grid cells in autumn had overlap > 0.75, but the majority of overlap values were ≤ 0.4 in autumn and ≤ 0.1 in winter). Possibly, the spatial match is stronger at a different scale. A process should be observed at the smallest scale where a driving variable affects the outcome of the process (the process scale, [62]). For the cod-capelin interaction, the process scale corresponds to the scale where cod or capelin can detect and respond to a change in the other’s density, which is likely at a much smaller scale than we could study with the data at hand. However, the behavioural response race between predator and prey would most likely result in negative predator-prey associations and a weaker spatial match at a finer scale [2, 53].

Distribution of alternative prey could also influence the spatial distribution of cod, but was outside the scope of this paper. Johannesen et al. [63] studied cod-prey interaction in autumn (2004–2009), including capelin, amphipods (Themisto sp.), herring (*Clupea harengus*), shrimp (*Pandalus borealis*) and polar cod (*Boreogadus saida*) as alternative prey. The only consistently positive relationship between both cod diet and cod distribution and prey density was found for capelin. The strongest candidate as important alternative prey during autumn is polar cod, which is found in the cold waters of the northern Barents Sea.

A possible explanation for the weak spatial match is that it is not necessary for cod to track the highest densities of capelin. Considering that it takes several days for a cod to digest a stomach full of capelin in the cold waters of the Barents Sea [64], the time and energy required to track the highest capelin densities is perhaps better spent digesting while remaining in an area of intermediate capelin density. In the Newfoundland cod-capelin system, no evidence of aggregative response of cod to capelin was found at scales up to 10 km or 100 km [53, 65] (but see also [3]). Using bioenergetic calculations, Horne and Schneider [65] argued that cod did not need to actively track capelin since the prey encounter rate was higher than the digestive rate at the observed capelin density. Constraints on cod digestion, in turn influenced by temperature, may therefore reduce predation when capelin is above a certain density threshold and cod is satiated. Finally, we considered horizontal overlap only, but diurnal vertical migration by capelin [66] may affect cod’s ability to efficiently track capelin.

### The relative role of temperature and abundance on spatial distributions and overlap

**Fig 10:**
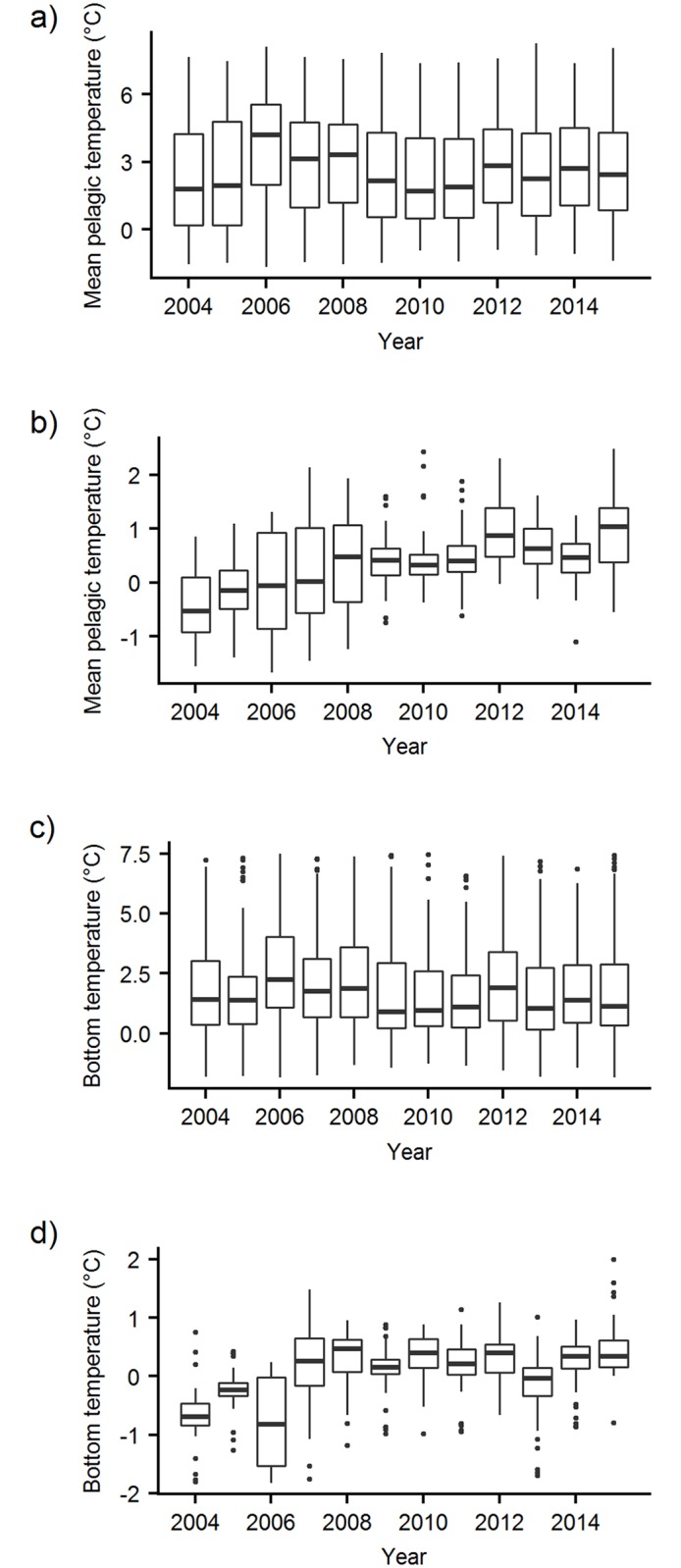
Autumn temperatures. Ecosystem survey measurements of A) mean pelagic temperature (50–200 m) in the entire study area, B) mean pelagic temperature in the north-eastern area (east of 40 E, north of 75 N), C) bottom temperature in the entire study area, D) bottom temperature in the north-eastern area, throughout the study period.

Effects of the physical environment and of species abundance on distributions have been difficult to disentangle in other cod-capelin systems (e.g., [8]), and the Barents Sea is no exception. The large-scale distributions of both cod and capelin have been related to ocean temperature, as well as stock size [13, 16, 17, 63, 67–71]. Here we attempted to separate the two by accounting for both stock biomass and temperature in our models. We found strong effects of stock biomass on both cod and capelin distributions in autumn, which in particular affected the strength and spatial pattern of overlap between immature cod and capelin (Fig 8 a, upper panels), but more moderate effects of temperature. Similarly, in the Baltic cod population, stock size had a stronger effect on local cod density than hydrography [72]. While our study period was characterised by high and relatively stable temperatures [26], the stock sizes of cod and capelin varied greatly (Fig 1). Our results are thus consistent with the lack of inter-annual contrast in the temperature data. However, there was one exception to the stable autumn temperatures observed throughout the study period. In the north-eastern area, local temperature increased with almost 1°C early in the study period (Fig 10), and we saw strong positive local effects of temperature on both cod and capelin densities in the same area (Fig 5A–5C). Together with the local effects of stock biomass, this explained the north-eastward shift in the overlap area between both cod components and capelin (Fig 8A and S4 Appendix).

In winter, influence of both temperature and stock biomass on the overlap was more variable than in autumn, since these factors either did not have strong effects on local species densities, had opposite effects on cod and capelin densities, or did not affect species densities in the main overlap areas. It is likely that the diverging behavioural motivation between immature and mature capelin, and the lack of a strong quantitative index of capelin density contribute to masking any clear signals in the winter data.

### Implications for stock assessment and future work

Due to the difficulty of monitoring and estimating the capelin stock in winter immediately prior to the fishing season, the capelin stock prediction model used in the assessment simulates the stock six months into the future from the time of monitoring in autumn to terminated spawning. Predation by immature cod on mature capelin is explicitly modelled for the first three months of the year, while interactions between other cod-capelin components are ignored [22, 23]. Our results on winter overlap (Fig 8B) demonstrate that interactions between other cod-capelin components may be important (see also [23, 24, 55]), emphasising that assumptions in stock prediction models that rely on an understanding of predator-prey interactions in highly dynamic systems should be tested regularly. The analytic framework applied here can be used to analyse and assess predator-prey overlap as part of regular monitoring.

## Supporting information

S1 Appendix Soap film smoother construction. (PDF)

S2 Appendix Wild bootstrap code and results. (HTML)

S3 Appendix Smooth functions of sun height and survey day. (PDF)

S4 Appendix Predicted overlap by year. (PDF)
