# Supplementary material for: Seasonal dynamics of spatial distributions and overlap between Northeast Arctic cod (Gadus morhua) and capelin (Mallotus villosus) in the Barents Sea
Source: PLoS One. 2018 Oct 16;13(10):e0205921. doi: 10.1371/journal.pone.0205921 (PMC6191152; doi:10.1371/journal.pone.0205921)
Supplement: S1 Appendix — (PDF) [file pone.0205921.s001.pdf]

Construction of a border and internal knots for the soap film smoother

Johanna Fall

August, 2018

Consider a marine species distributed in a region that contains a physical boundary, such as an island. Species density is naturally zero on land, and we can conceive of a situation where the density on one side of the island is very different from that on the other side. When using conventional smoothing splines, such as the default thin-plate regression splines in GAM (Wood 2003), the smooth functions are fitted over the entire x-y-space defined by the range of our sampling locations. Since the model does not know that this space contains an island, there is a risk of it fitting a non-zero density on the land area, and the model is also likely to fit very similar densities on all sides of the island since smooth functions must change gradually with the Euclidian distance (Miller and Wood 2014). These issues can lead to prediction errors. One way to account for geographical boundaries in the GAM is to use the soap film smoother (Wood et al. 2008, see also Miller and Wood 2014 for an alternative approach). The idea is to restrict the smooth function by fitting it within a certain boundary, much like the shape of a soap bubble is determined by the bubble wand. In our case, we used the coastline of Norway and Russia, as well as the contours of the main island groups within the Barents Sea (Svalbard, Franz Josef Land, and Novaya Zemlya) as our boundary.

Two things need to be specified in order to construct a soap film smoother:

1. The boundary within which to fit the smooth function, which can include holes for islands.
2. A number of knots with grid spacing delta, inside this boundary area. These knots are not the same as the ones specified with the 'k' argument in the GAM; those knots determine the number of basis functions that will be solved when fitting a smooth term, while the interior knots determine the accuracy of those functions.

To check that 1. and 2. have been set up correctly, this function is helpful [Miller 2015, https://github.com/dill/soap_checker].

The following code is based on the one proposed by Simpson (2016 [http://www.fromthebottomoftheheap.net/2016/03/27/soap-film-smoothers/#fn9]), see also Augustin et al. 2013), extended to include the construction

of a complex border from a shapefile of the Barents Sea. Libraries required are: *raster*, *rgdal*, *sp*, *rgeos*, *rmapshaper*,*broom*, *dplyr*, and *mgcv*.

First, we construct the boundary, or the soap bubble wand:

```
#This is a shapefile that contains the coastline and islands of interest
#The readOGR function converts the shapefile into a SpatialPolygonsDataFrame
polygons <- readOGR(dsn = 'C:/JF Library/WP2/WP2 analyses/Shapefiles BS/50m', layer = 'land_BS')
```

```
## OGR data source with driver: ESRI Shapefile
## Source: "C:\JF Library\WP2\WP2 analyses\Shapefiles BS\50m", layer: "land_BS"
## with 51 features
## It has 2 fields
```

```
#This shapefile describes the 500 m isobath. This will be a part of the outer border as a way of restri
#study area to the Barents Sea shelf.
iso500 <- readOGR(dsn = 'C:/JF Library/WP2/WP2 analyses/Shapefiles BS', layer = 'Kontur500_2')
```

```
## OGR data source with driver: ESRI Shapefile
## Source: "C:\JF Library\WP2\WP2 analyses\Shapefiles BS", layer: "Kontur500_2"
## with 423 features
## It has 1 fields
```

```
par(mar=c(0,0,0,0))
plot(polygons, col = "blue")
plot(iso500, add = T)
```

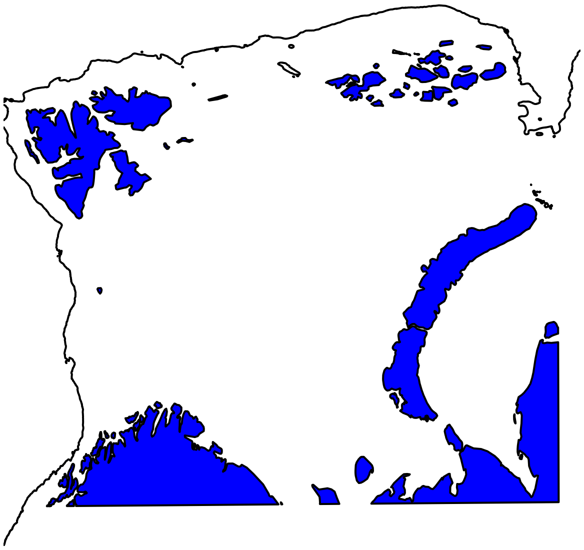

The outlines need to be simplified since they are too complicated for the soap smoother to handle, and the isobath data extends outside the study area. The level of complexity that can be used in the GAM is a matter of trial and error. Here, I settle on keeping 10 % of the original data for the islands and coast, and 1 % for the 500 m isobath, which contained a lot of data. This way a few small islands and fine contour details are lost, but we keep the most important features.

```
#Simplify the outline of the land masses
ocean.simp <- ms_simplify(polygons, keep = 0.1, keep_shapes = FALSE, explode = TRUE)
```

```
iso.simp <- ms_simplify(iso500, keep = 0.01, keep_shapes = FALSE, explode = TRUE)
```

```
par(mfrow = c(2,2), mar=c(0,0,1,0))
plot(polygons, col = "blue", main = "Original")
plot(ocean.simp, col = "blue", main = "10 %")
plot(iso500, col = "blue", main = "Original")
plot(iso.simp, col = "blue", main = "1 %")
```

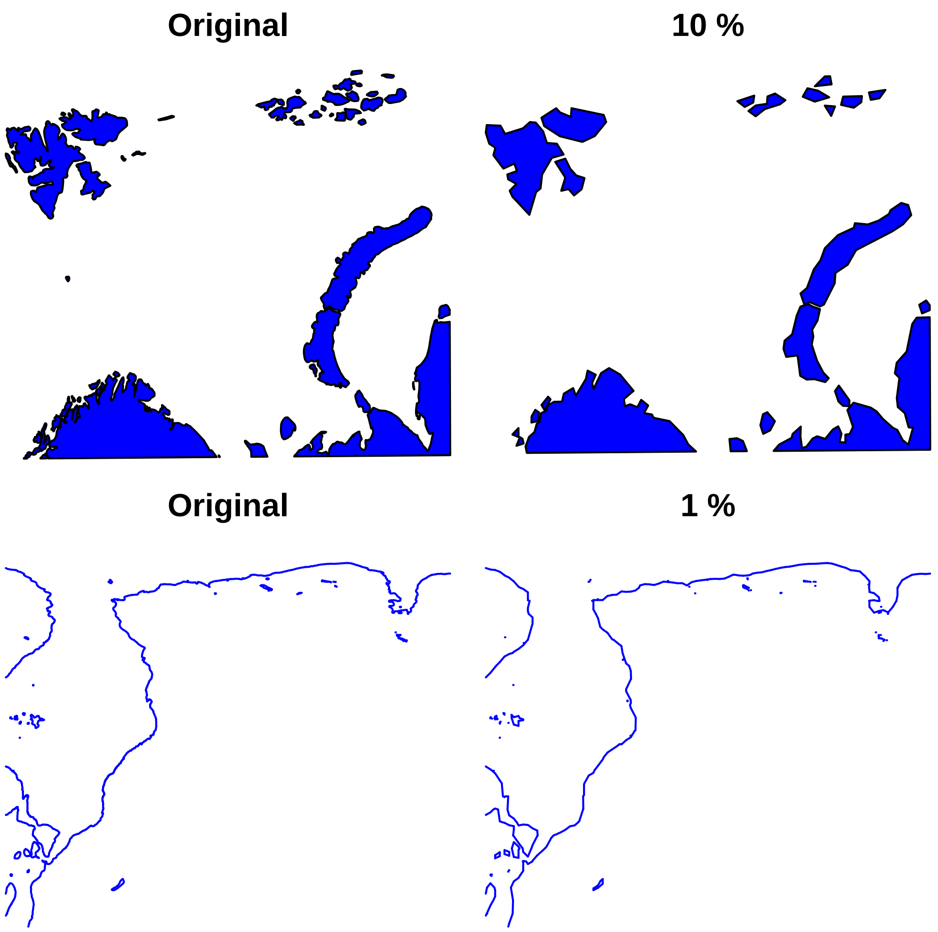


```
## null device
##           1
```

I then convert the coordinates to stereographic projection, centered in the approximate middle of our study area. This coordinate system accounts well for the curvature of the earth at high latitudes.

```
#Project in polar stereographic coordinate system
land <- spTransform(ocean.simp, "+proj=stere +lat_0=75 +lon_0=35 +datum=WGS84 +units=m")
isobath <- spTransform(iso.simp, "+proj=stere +lat_0=75 +lon_0=35 +datum=WGS84 +units=m")
```

Because the isobath data that describes the 500 m depth contour is in a SpatialLines format and not polygons, some processing is needed to get everything ready for clipping out the islands from the ocean.

```
#This file contains a simple boundary encircling the isobath,
#from which we will clip out the contours of the isobath
boundary <- read.table("C:/JF Library/WP2/WP2 analyses/Soap smooths/boundary clipping polygon autumn.tx
```

```r
                        header = TRUE)


#Convert it to a polygon
p <- Polygon(boundary)
ps <- Polygons(list(p),1)
sps <- SpatialPolygons(list(ps))
proj4string(sps) <- CRS("+proj=stere +lat_0=75 +lon_0=35 +datum=WGS84 +units=m")
par(mar=c(0,0,0,0))
plot(isobath)
plot(sps, add = TRUE)
```

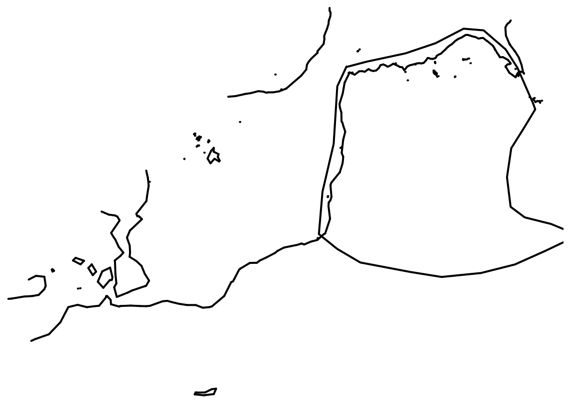

```r
#Clip out the isobath from the boundary polygon:
# intersect the line with the polygon
lpi <- gIntersection(sps, isobath)
# create a very thin polygon buffer of the intersected line
blpi <- gBuffer(lpi, width = 0.000001)
# split using gDifference
dpi <- gDifference(sps, blpi)
# convert polygon to data frame
dpi.df <- tidy(dpi)
# select the piece of interest
dpi.df <- dpi.df[dpi.df$piece == 1, ]
```

```
#Make new polygon from the fixed border
dpi.list <- list(x = dpi.df$long, y = dpi.df$lat)
p <- Polygon(dpi.list)
ps <- Polygons(list(p),1)
sps <- SpatialPolygons(list(ps))
proj4string(sps) <- CRS("+proj=stere +lat_0=75 +lon_0=35 +datum=WGS84 +units=m")
plot(land, col = "green")
plot(sps, add = TRUE)
```

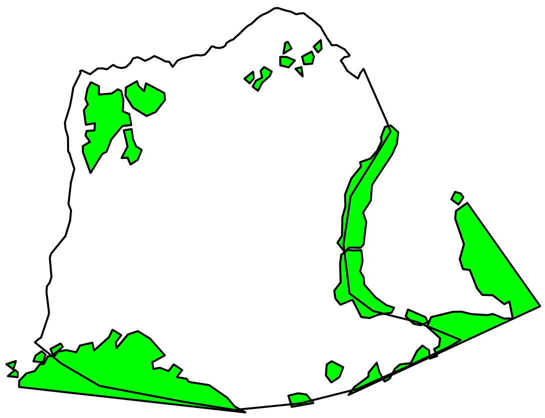

```
#Simplify the outer border a little more
sps.simp <- ms_simplify(sps, keep = 0.30, keep_shapes = TRUE, explode = TRUE)

#Add a buffer and clip out the land masses from the ocean layer
tst <- gBuffer(sps, byid=TRUE, width=0)
tst2 <- gBuffer(land, byid=TRUE, width=0)


ocean.aut <- erase(tst, tst2)


#Check so the geometry of this new polygon is valid, and plot it
rgeos::gIsValid(ocean.aut)
```

```
## [1] TRUE
```

```r
plot(ocean.aut, col = "blue")
```

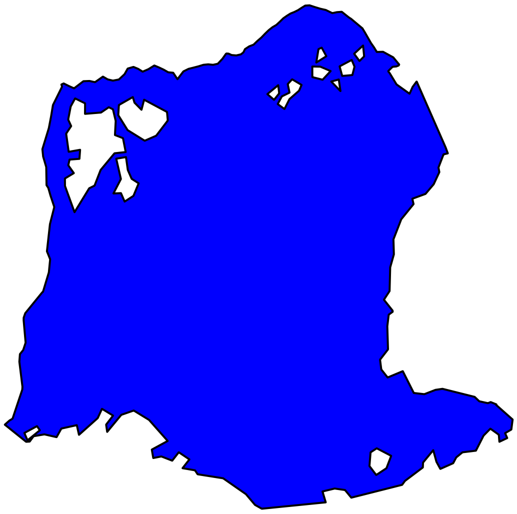

```r
#Convert the spatial object to a data frame
ocean.xy.aut <- tidy(ocean.aut)


#express coordinates in nautical miles instead of metres to match the data
ocean.xy.aut$long <- ocean.xy.aut$long/1852
ocean.xy.aut$lat <- ocean.xy.aut$lat/1852
ocean.xy.aut <- ocean.xy.aut %>% rename(x = long, y = lat)
```

This is our boundary polygons that contain area we want to fit the soap film smooths in.

Next we define the interior knots. Following the method of Simpson (2016), I use the extent of the data to define a regular grid over the study area. According to Wood (2008), the spacing of the interior knots should generally be smaller then the distance between the observations. However, since our study area is so large this gives us a very large number of knots that slows down the gam-function considerably. Such a fine resolution also leads to the spatial term capturing previously robust patterns of the other covariates, suggesting that we are overfitting the model. Therefore, I settle on using a knot spacing of 40 nm, slightly larger than the ~35 nm interstation distance of the autumn data.

```r
#Load the data
autdat <- read.table("C:/JF Library/WP2/WP2 analyses/Eco/eco trawl clipped for soap.txt",
                     header = TRUE)
```

```r
#Make knots from the geographical extent of the observations
N <- floor((abs((max(autdat$x)-min(autdat$x)))/40))
gx <- seq(min(autdat$x), max(autdat$x), length.out = N)
gy <- seq(min(autdat$y), max(autdat$y), length.out = N)
gp <- expand.grid(gx, gy)
names(gp) <- c("x","y")
plot(gp$x, gp$y)
```

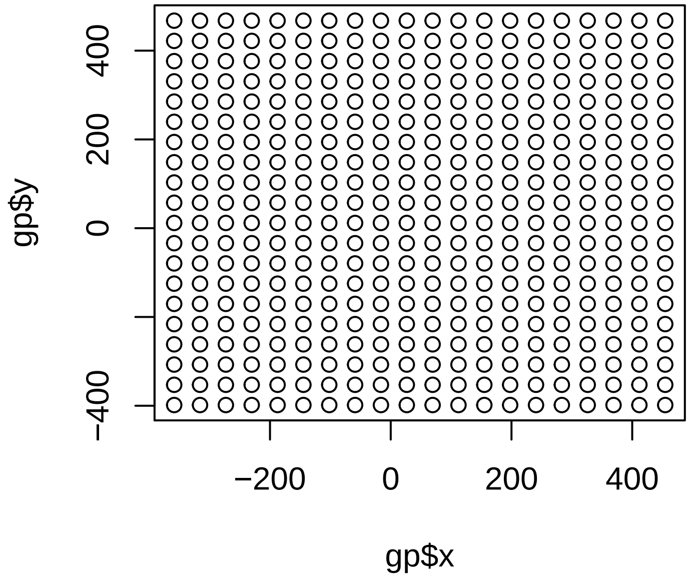


```r
#The GAM needs the border coordinates as a list of lists,
#where each list describes one border segment or island:
oceancoords <- ocean.xy.aut %>% dplyr::select(x,y,piece)
names(oceancoords) <- c("x", "y", "piece")
borderlist <- split(oceancoords, oceancoords$piece)
names(borderlist)
```

```
##  [1] "1"  "2"  "3"  "4"  "5"  "6"  "7"  "8"  "9"  "10" "11" "12" "13"
```

```r
border.aut <- lapply(borderlist, `[`, c(1,2))
nr <- seq(1,13)
```

```r
border.aut <- lapply(nr, function(n) as.list.data.frame(border.aut[[n]]))

#We can now use the inSide function from mgcv to select knots that are inside the border
knots <- gp[with(gp, inSide(bnd = border.aut, x, y)), ]
names(knots) <- c("x", "y")

#And then check that the border and knots are in order with the soap_check function
source("C:/JF Library/WP2/WP2 analyses/Soap smooths/soap_check.R")
par(mar=c(0,0,1,0))
soap_check(bnd = border.aut, knots = knots)
```

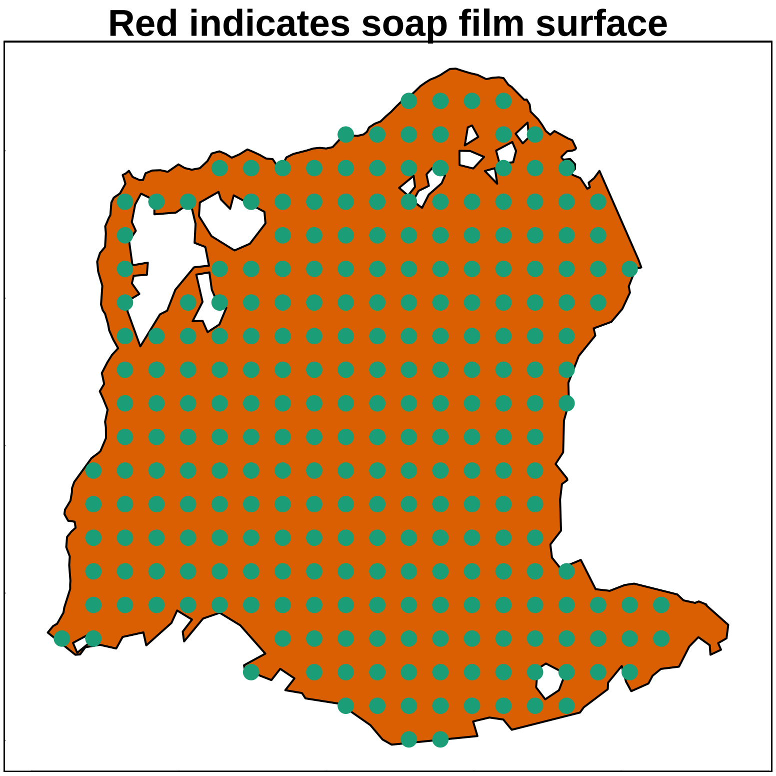


```
## [1] TRUE
```

This seems to be in order, but infortunately the algorithm that GAM uses to check if the knots are inside the boundary is not the same as the one used by the InSide function. It is therefore often necessary to adjust the location of some knots manually.

```r
#Load a table of adjusted knots
knots.aut <- read.table("C:/JF Library/WP2/WP2 analyses/Soap smooths/knots autumn 40 nm.txt",
```

```
                        header = T)
```

Finally, we must make sure that all the data we want to analyse is inside the boundary area.

```
#Make sure there is no data outside the boundary defined by the ocean polygon
#Convert data to spatial
autdat.sp <- autdat
autdat.sp$x <- autdat.sp$x*1852 #convert back from nmi to m
autdat.sp$y <- autdat.sp$y*1852
autdat.sp <- SpatialPointsDataFrame(autdat.sp[,c(6,5)], autdat.sp,
                                    proj4string = CRS("+proj=stere +lat_0=75
                                                  +lon_0=35 +datum=WGS84 +units=m"))

#Clip the data to match the ocean polygon - this removes a few observations from a year
#when the ice conditions allowed sampling north-east of Franz Josef Land.
data.clipped <- crop(autdat.sp, ocean.aut)
par(mar=c(0,0,1,0))
plot(data.clipped)
plot(ocean.aut,add=T)
```

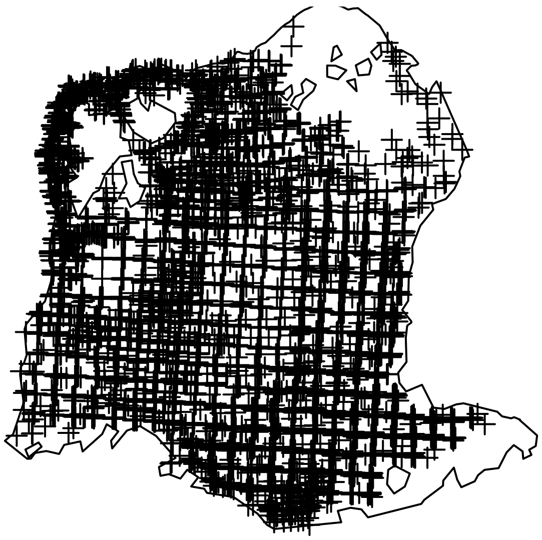

```
autdat <- data.frame(data.clipped)
autdat$x <- autdat$x/1852 #convert back to nmi
autdat$y <- autdat$y/1852
```

Now we are ready to fit a GAM with soap film smooths. I will illustrate this with a model that describes the density of immature cod in autumn as a function of a spatial term fitted with the soap basis, in addition to conventional smooth terms of bottom depth, bottom temperature, and sunheight and survey day to correct for timing of sampling. This model takes a bit longer to run than one with a conventional smoothing basis, but we can control this to some extent by reducing the number of internal knots that is supplied in the 'knots = knots.aut' argument (as constructed above). The soap basis is specified in the 'bs = "so"' argument, and the list of border coordinates in the 'xt = list(bnd = border)' argument. We also put a constraint of 20 on the basis dimension ("k") of the spatial smooth to reduce runtime and avoid overfitting the spatial field.

```
imm.aut.soap <- gam(cod.imm ~ s(bio_cod, k = 5) +
                      s(x, y, k = 20, bs = "so", xt = list(bnd = border.aut)) +
                      s(sunheight, k = 5) + s(s.day, k = 5) +
                      s(b_depth, k = 5) + s(b_temp, k = 5),
                    data = autdat, family = tw(), method = "REML", knots = knots.aut)
```

```
summary(imm.aut.soap)
```

```
##
## Family: Tweedie(p=1.628)
## Link function: log
##
## Formula:
## cod.imm ~ s(bio_cod, k = 5) + s(x, y, k = 20, bs = "so", xt = list(bnd = border.aut)) +
##     s(sunheight, k = 5) + s(s.day, k = 5) + s(b_depth, k = 5) +
##     s(b_temp, k = 5)
##
## Parametric coefficients:
##             Estimate Std. Error t value Pr(>|t|)
## (Intercept)   6.8459     0.0801   85.47   <2e-16 ***
## ---
## Signif. codes:  0 '***' 0.001 '**' 0.01 '*' 0.05 '.' 0.1 ' ' 1
##
## Approximate significance of smooth terms:
##                   edf  Ref.df       F  p-value
```

```
## s(bio_cod)      3.308   3.743  26.526  < 2e-16 ***
## s(x,y)        171.948 250.000   8.168  < 2e-16 ***
## s(sunheight)    3.132   3.618  30.101  < 2e-16 ***
## s(s.day)        3.161   3.634   4.620  0.00159 **
## s(b_depth)      3.827   3.970 113.427  < 2e-16 ***
## s(b_temp)       3.663   3.924  15.587 1.23e-12 ***
## ---
## Signif. codes:  0 '***' 0.001 '**' 0.01 '*' 0.05 '.' 0.1 ' ' 1
##
## R-sq.(adj) =  0.144   Deviance explained = 49.2%
## -REML =  30742  Scale est. = 23.134    n = 4644
```

```
par(mar=c(1,1,1,1))
plot(imm.aut.soap, select = 2)
```

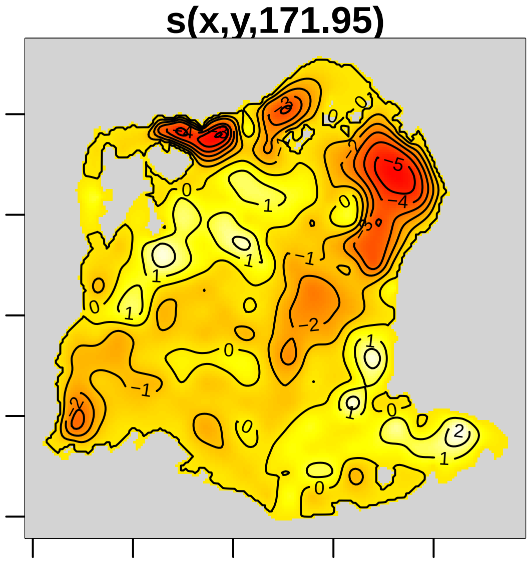

Let us compare this model with one fitted with the usual “tp” smoothing basis. Here we put no constraint on the s(x,y)-term to let the gam-function find the best fit of this term, i.e., the best possible model with tp-basis.

```
imm.aut.tp <- gam(cod.imm ~ s(bio_cod, k = 5) + s(x, y) +
                    s(sunheight, k = 5) + s(s.day, k = 5) +
                    s(b_depth, k = 5) + s(b_temp, k = 5),
                  data = autdat, family = tw(), method = "REML")
```

```r
summary(imm.aut.tp)
```

```
##
## Family: Tweedie(p=1.653)
## Link function: log
##
## Formula:
## cod.imm ~ s(bio_cod, k = 5) + s(x, y) + s(sunheight, k = 5) +
##     s(s.day, k = 5) + s(b_depth, k = 5) + s(b_temp, k = 5)
##
## Parametric coefficients:
##             Estimate Std. Error t value Pr(>|t|)
## (Intercept)  6.67663    0.02311   288.9   <2e-16 ***
## ---
## Signif. codes:  0 '***' 0.001 '**' 0.01 '*' 0.05 '.' 0.1 ' ' 1
##
## Approximate significance of smooth terms:
##                 edf Ref.df       F p-value
## s(bio_cod)    2.667  3.188  25.620 < 2e-16 ***
## s(x,y)       27.802 28.901  37.665 < 2e-16 ***
## s(sunheight)  3.135  3.624  27.737 < 2e-16 ***
## s(s.day)      2.628  3.164   4.031 0.00586 **
## s(b_depth)    3.797  3.974 174.393 < 2e-16 ***
## s(b_temp)     3.557  3.898  38.870 < 2e-16 ***
## ---
## Signif. codes:  0 '***' 0.001 '**' 0.01 '*' 0.05 '.' 0.1 ' ' 1
##
## R-sq.(adj) =  0.0887   Deviance explained = 38.7%
## -REML =  30944  Scale est. = 22.656    n = 4644
```

```r
par(mfrow = c(1,2), mar=c(1,1,1,1))
vis.gam(imm.aut.soap, view = c("x", "y"), plot.type = "contour")
vis.gam(imm.aut.tp, view = c("x", "y"), plot.type = "contour")
```

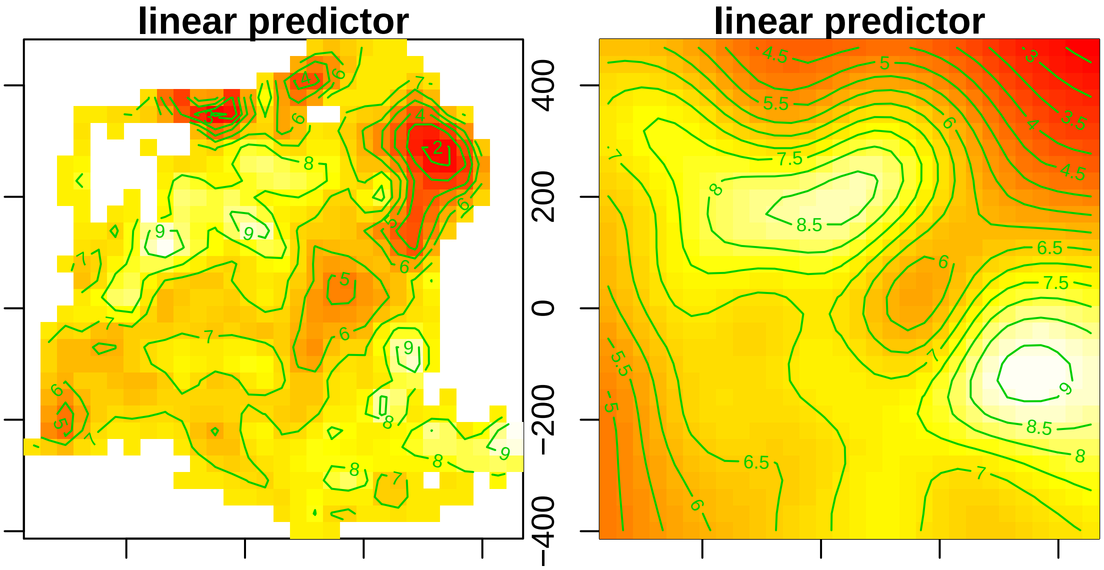

It is immediately apparent that the tp-model predicts high densities in a land area in the south east, since we have sampled high cod densities close to the coast there. Though it is more complex, the soap model has higher explanatory power than the tp-model, and lower AIC.

```
##                     df      AIC
## imm.aut.soap 195.08419 61197.12
## imm.aut.tp    48.91781 61901.69
```

Choosing the number of internal knots

As mentioned above, choosing the number and placement of internal knots can be tricky and time consuming. It is also crucial to the fit of the model, since these knots determine the spatial resolution of the smooth function(s). This sets the soap film smoother apart from other basis functions where it is often enough to specify them directly in the call to gam without previous preparation. It is therefore important to explore the effect of changing the number and placement of the knots in order to find a reasonable trade-off between resolution and computational time; the latter is particularly crucial for highly complex models. For this analysis, we got a similar general pattern of distribution if we removed up to 20% of the knots we had settled on using, but relevant finer features were lost. Thus, when using the soap film smoother, consider how fine features are relevant to the objectives of your study and how this will affect the runtime of your models.

References

Augustin, N. H., Trenkel, V. M., Wood, S. N., & Lorance, P. (2013). Space-time modelling of blue ling for fisheries stock management. Environmetrics, 24(2), 109-119. doi:10.1002/env.2196

Miller, D. L., and Wood, S. N. 2014. Finite area smoothing with generalized distance splines. Environmental and Ecological Statistics, 21: 715-731. doi:10.1007/s10651-014-0277-4

Miller, D. L. 2015, August 15. "Check whether a soap film smoother boundary and knots make sense".*Github.com.* Retrieved August 29, 2017, from https://github.com/dill/soap_checker

Simpson, G. 2016, March 27. "Soap-film smoothers & lake bathymetries". *fromthebottomoftheheap.net*. Retrieved August 29, 2017, from http://www.fromthebottomoftheheap.net/2016/03/27/soap-film-smoothers/#fn9

Wood, S. N., Bravington, M. V., & Hedley, S. L. (2008). Soap film smoothing. Journal of the Royal Statistical Society: Series B (Statistical Methodology), 70(5), 931-955. doi:10.1111/j.1467-9868.2008.00665.x

See also: [https://people.maths.bris.ac.uk/~sw15190/talks/soap-talk.pdf]
