# Supplementary material for: Seasonal dynamics of spatial distributions and overlap between Northeast Arctic cod (Gadus morhua) and capelin (Mallotus villosus) in the Barents Sea
Source: PLoS One. 2018 Oct 16;13(10):e0205921. doi: 10.1371/journal.pone.0205921 (PMC6191152; doi:10.1371/journal.pone.0205921)
Supplement: S2 Appendix — (HTML) [file pone.0205921.s002.html]

Wild bootstrap on the capelin acoustic data


# Wild bootstrap on the capelin acoustic data

#### *Johanna Fall and Lorenzo Ciannelli*

#### *September 21, 2018*

## Example code for winter acoustic data

```
####### Wild bootstrap on the capelin acoustic data after Llope et al. (2009) ###########

#Fit a gam to the data. Since the fitting of soap smooths is too slow to do 1000 iterations in 
#a reasonable time, we fit a model with the default tp-basis. This model has similar residual 
#autocorrelation as the corresponding model with soap basis. We will run the bootstrap 
#separately for each year and therefore do not include year (or biomass which is correlated 
#with year) as a covariate.
gam1 <- gam(capelin ~ s(x, y, k = 20) +
              s(sunheight, k = 5) + s(s.day, k = 5) + s(b_depth, k = 5) + #using same k as in the soap models
              s(p_temp, k = 5), data = windat, family = tw())
# summary(gam1)
# Family: Tweedie(p=1.607) 
# Link function: log 
# 
# Formula:
#   capelin ~ s(x, y, k = 20) + s(sunheight, k = 5) + s(s.day, k = 5) + 
#   s(b_depth, k = 5) + s(p_temp, k = 5)
# 
# Parametric coefficients:
#   Estimate Std. Error t value Pr(>|t|)    
#   (Intercept)    2.292      0.050   45.85   <2e-16 ***
#   ---
#   Signif. codes:  0 ‘***’ 0.001 ‘**’ 0.01 ‘*’ 0.05 ‘.’ 0.1 ‘ ’ 1
# 
# Approximate significance of smooth terms:
#   edf Ref.df      F  p-value    
#   s(x,y)       18.322 18.948 72.838  < 2e-16 ***
#   s(sunheight)  1.005  1.010 33.023 9.09e-09 ***
#   s(s.day)      3.856  3.987  9.015 1.48e-06 ***
#   s(b_depth)    3.083  3.595  1.892   0.0838 .  
#   s(p_temp)     3.902  3.994 36.259  < 2e-16 ***
#   ---
#   Signif. codes:  0 ‘***’ 0.001 ‘**’ 0.01 ‘*’ 0.05 ‘.’ 0.1 ‘ ’ 1
# 
# R-sq.(adj) =  0.168   Deviance explained = 50.2%
# -REML = 9052.9  Scale est. = 19.718    n = 3994

#Extract the response residuals for calculating CIs. We use the response residuals since they 
#are straightforward to add back to the predicted values, 
#while still preserving the autocorrelation in the data
cap.res.response <- residuals(gam1, type = "response")

#Extract scaled pearson residuals (scaled to the variance of the model fit) for calculating p-values
#we use the scaled residuals here to get more accurate p-values, since they reflect the true error of the model
cap.res.scaled<-residuals(gam1,type='scaled.pearson')

##P-values and CI
years<-unique(windat$year)

#Create matrices to iterate over, one for the smooth terms (and one for the parametric terms if any)
#Here we will store the F-statistic for each term
ps.boot<-matrix(NA,ncol=5,nrow=1000) #one column for each smooth term in model
# pp.boot<-matrix(2000,ncol=1,nrow=1000)*NA #if there are parametric terms in model

#Create matrices for each covariate where we will store the predicted values for each iteration
cs.pos<-matrix(nrow(windat)*1000,ncol=1000,nrow=nrow(windat))*NA #number of iterations
cs.depth<-cs.pos
cs.sunheight<-cs.pos
cs.sday <- cs.pos
cs.temp <- cs.pos

#Create new variables for the scaled residuals that will be randomly assigned
#a new sign for each iteration
windat$res.scaled<-NA
windat$res.response<-NA

#Number of iterations in the bootstrap
b< - 1000 

#Run the bootstrap (took approximately 7 hours for this model)
for(i in 1:b){
  for(j in 1:length(years)){
    #randomly change sign of all residuals within a year:
    windat$res.scaled[windat$year==years[j]]<-
      cap.res.scaled[windat$year==years[j]]*sample(c(-1,1),1)
        windat$res.response[windat$year==years[j]]<-
      cap.res.response[windat$year==years[j]]*sample(c(-1,1),1)
    #add residuals to model fit
    windat$newy[windat$year==years[j]]<- 
      gam1$fitted.values[windat$year==years[j]]+windat$res.response[windat$year==years[j]]
    #make sure there are no negative predictions (capelin must be >=0 - the response in untransformed
    #in a model with Tweedie distribution)
    windat$newy[windat$year==years[j] & windat$newy < 0] <- 0} 
  #fit a model to the scaled residuals (whose sign were randomly flipped), 
  #these models will be used to calculate p-values
  boot.gam<-gam(res.scaled ~ s(x, y, k = 20) + s(sunheight, k = 5) + s(s.day, k = 5) + s(b_depth, k = 5) +
                  s(p_temp, k = 5), data = windat)
  #add the response residuals to the data and fit a new model, there models will be used to get 
  #the upper and lower CI-limits 
  boot.gam.data<-gam(newy ~ s(x, y, k = 20) + s(sunheight, k = 5) + s(s.day, k = 5) + s(b_depth, k = 5) +
                       s(p_temp, k = 5), data = windat, family = tw()) 
  #Store predictions for the data +/- residuals
  cs.posW[,i]<-predict(boot.gam.data,type='terms')[,1] 
  cs.sunheightW[,i]<-predict(boot.gam.data,type='terms')[,2]
  cs.sdayW[,i]<-predict(boot.gam.data,type='terms')[,3]
  cs.depthW[,i]<-predict(boot.gam.data,type='terms')[,4]
  cs.tempW[,i]<-predict(boot.gam.data,type='terms')[,5]
  #Store the table of smooth term fits for residuals model
  ps.bootW[i,]<-summary(boot.gam)$s.table[,3] 
}

#Calculate new p-values based on the mean F statistic over the 1000 runs. The new p-value is the percentage
#(probability) of the bootstrapped F or t values ≥ observed ones (original gam)
p.vals.posW<- sum(1*(ps.bootW[,1]>=summary(gam1)$s.table[1,3]))/b
#0
p.vals.sunW<- sum(1*(ps.bootW[,2]>=summary(gam1)$s.table[2,3]))/b
#0.02
p.vals.sdayW<- sum(1*(ps.bootW[,3]>=summary(gam1)$s.table[3,3]))/b
#0.004
p.vals.depthW<- sum(1*(ps.bootW[,3]>=summary(gam1)$s.table[4,3]))/b
#0.327
p.vals.tempW<- sum(1*(ps.bootW[,3]>=summary(gam1)$s.table[5,3]))/b
#0

#Calculate CI for each smooth term

#1. calculate the mean predicted value (smooth contribution) for each data point across the 1000 iterations.
mean.sunW<-apply(cs.sunheightW,1,mean)  
#2. and the lower bounds of the 95 % CI
low.sunW<-apply(cs.sunheightW,1,function(x)quantile(x,0.025)) 
#3. and the upper bounds
up.sunW<-apply(cs.sunheightW,1,function(x)quantile(x,0.975))

#Similarily, for the other covariates
mean.sdayW<-apply(cs.sdayW,1,mean)
low.sdayW<-apply(cs.sdayW,1,function(x)quantile(x,0.025))
up.sdayW<-apply(cs.sdayW,1,function(x)quantile(x,0.975))
mean.depthW<-apply(cs.depthW,1,mean)
low.depthW<-apply(cs.depthW,1,function(x)quantile(x,0.025))
up.depthW<-apply(cs.depthW,1,function(x)quantile(x,0.975))
mean.tempW<-apply(cs.tempW,1,mean)
low.tempW<-apply(cs.tempW,1,function(x)quantile(x,0.025))
up.tempW<-apply(cs.tempW,1,function(x)quantile(x,0.975))
```

## Plotting the bootstrapped smooth functions

### Winter

In this case, all covariates except depth remained significant (p < 0.05) after the bootstrap. This is in line with depth being near- or non-significant in some of our more complex candidate models (see main paper). Generally, the mean effects and confidence intervals were similar between the original model fit and the bootstrapped fit.

### Autumn

For the autumn data, the bootstrapped confidence intervals for survey day, depth, and temperature resulted in non-significant p-values. While depth was non-significant in some of the more complex candidate models, survey day and temperature were clearly important contributors to model fit when added as spatially variant terms in the candidate models. It is likely that the spatially variant formulations and the soap smoother then captured more of the residual autocorrelation in the models. For the autumn data, too, the confidence intervals and mean effects effects were nevertheless similar between the original model fit and the bootstrapped fit.

```
#Calculate new p-values based on the mean F statistic over the 1000 runs.
#The new p-value is the percentage (probability) of the bootstrapped F or t values ≥ observed ones (original gam)
p.vals.pos<- sum(1*(ps.boot[,1]>=summary(gam2)$s.table[1,3]))/1000
#0
p.vals.sun<- sum(1*(ps.boot[,2]>=summary(gam2)$s.table[2,3]))/1000
#0.029 
p.vals.jul<- sum(1*(ps.boot[,3]>=summary(gam2)$s.table[3,3]))/1000
#0.081 
p.vals.depth<- sum(1*(ps.boot[,3]>=summary(gam2)$s.table[4,3]))/1000
#0.123 
p.vals.temp<- sum(1*(ps.boot[,3]>=summary(gam2)$s.table[5,3]))/1000
#0.118
```
