# Supplementary material for: Seasonal dynamics of spatial distributions and overlap between Northeast Arctic cod (Gadus morhua) and capelin (Mallotus villosus) in the Barents Sea
Source: PLoS One. 2018 Oct 16;13(10):e0205921. doi: 10.1371/journal.pone.0205921 (PMC6191152; doi:10.1371/journal.pone.0205921)
Supplement: S3 Appendix — (PDF) [file pone.0205921.s003.pdf]

Supplement 2: Smooth functions of sun height and survey day

Here we show the one-dimensional smooth functions of sun height (degrees below or above the horizon) and survey day (sampling day relative to the first day of sampling across the study period for each season) from the *habitat models*, and spatially variant effects of survey day from the best *candidate models* for all components (immature cod, mature cod, capelin A = capelin sampled with acoustics, capelin T = capelin sampled with bottom trawl) from a) autumn and b) winter. Only effects retained in the models ($p < 0.05$) are shown, otherwise they are denoted n.s. (non-significant). For 1d-smooths, the y-axis represents the degree to which variation in the predictor can explain variation in the response, where the horizontal line at 0 represents a neutral effect. For the spatially variant effect, the contour lines show how the slope of the linear regression between species density and survey day varies in space. Yellow colours represent positive slopes and red colours negative slopes. Though there were clear spatial patterns, the changes in slopes were small.

a) Autumn

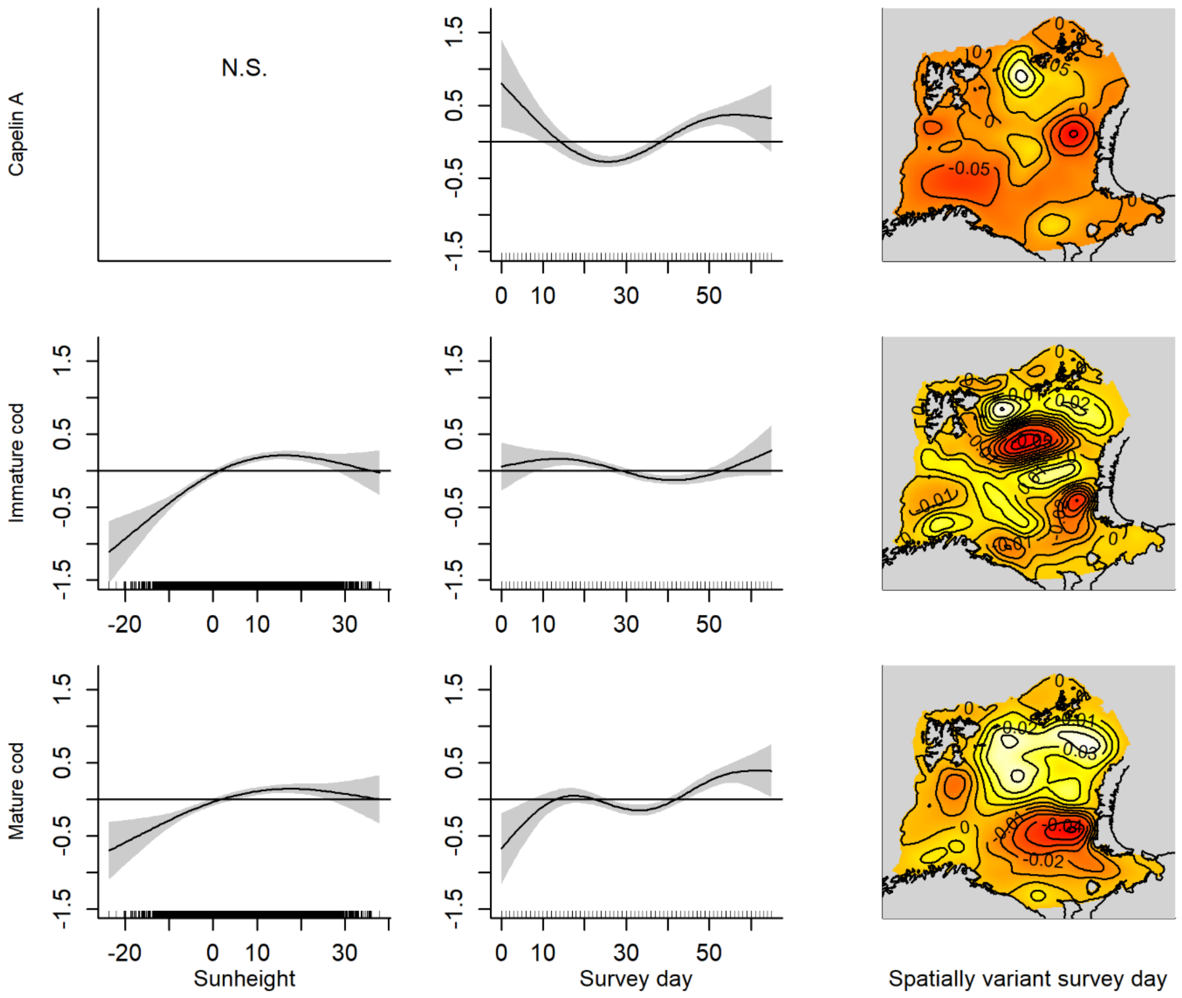

b) Winter

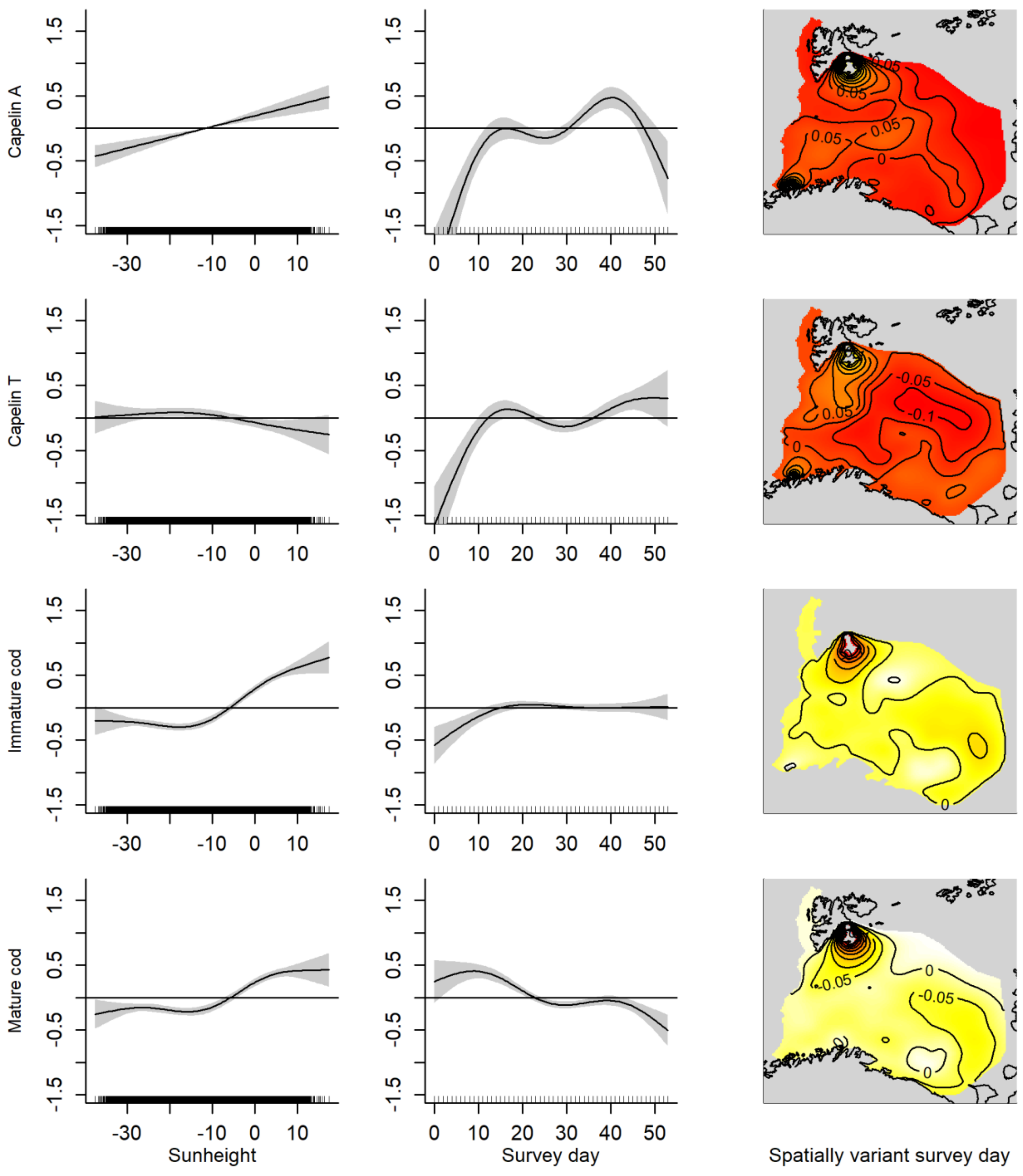

Capelin A
Capelin T
Immature cod
Mature cod
Sunheight
Survey day
Spatially variant survey day
