# Supplementary material for: Seasonal dynamics of spatial distributions and overlap between Northeast Arctic cod (Gadus morhua) and capelin (Mallotus villosus) in the Barents Sea
Source: PLoS One. 2018 Oct 16;13(10):e0205921. doi: 10.1371/journal.pone.0205921 (PMC6191152; doi:10.1371/journal.pone.0205921)

Supplement 3: Predicted overlap by year for each season and component pair

The overlap index O_{xyt} ranges from 0-1, where 1 means that the highest (year-specific) densities of cod and capelin are found in the grid cell. Because there were a few very high overlap values that made visualisation difficult, these were set to black color to enable a good representation of the variation in the main overlap range (values ≤ 0.4 in autumn and ≤ 0.1 in winter).The numbers in the bottom left corner of each panel represents the strength of correlation (kendall's tau) between the predicted densities of cod and capelin across the grid. This document contains the following figures:

a) Autumn: immature cod and acoustic capelin
b) Autumn: mature cod and acoustic capelin
c) Winter: immature cod and acoustic capelin
d) Winter: immature cod and trawl capelin
e) Winter: mature cod and acoustic capelin
f) Winter: mature cod and trawl capelin

a) Autumn: immature cod and acoustic capelin

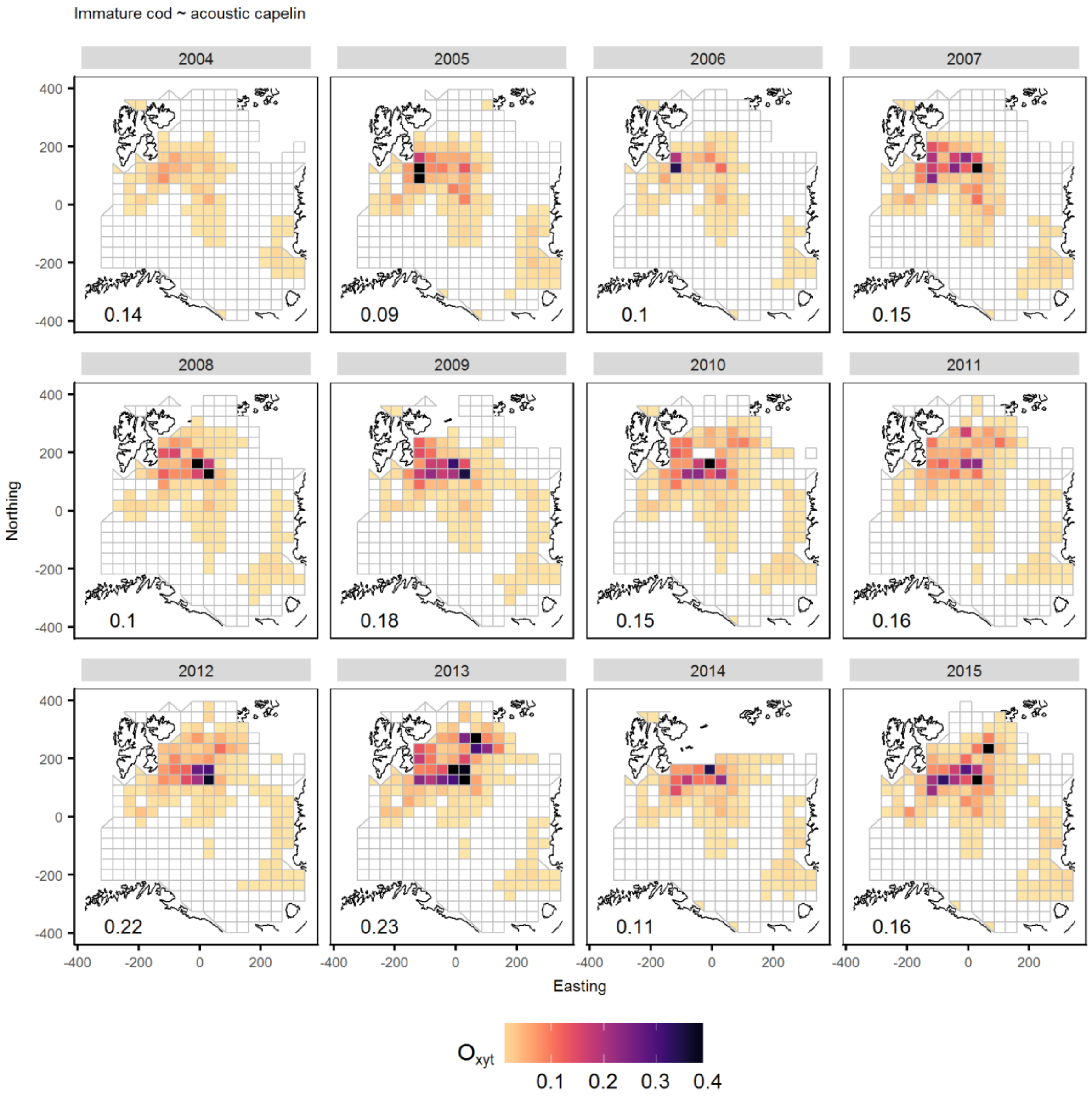

b) Autumn: mature cod and acoustic capelin

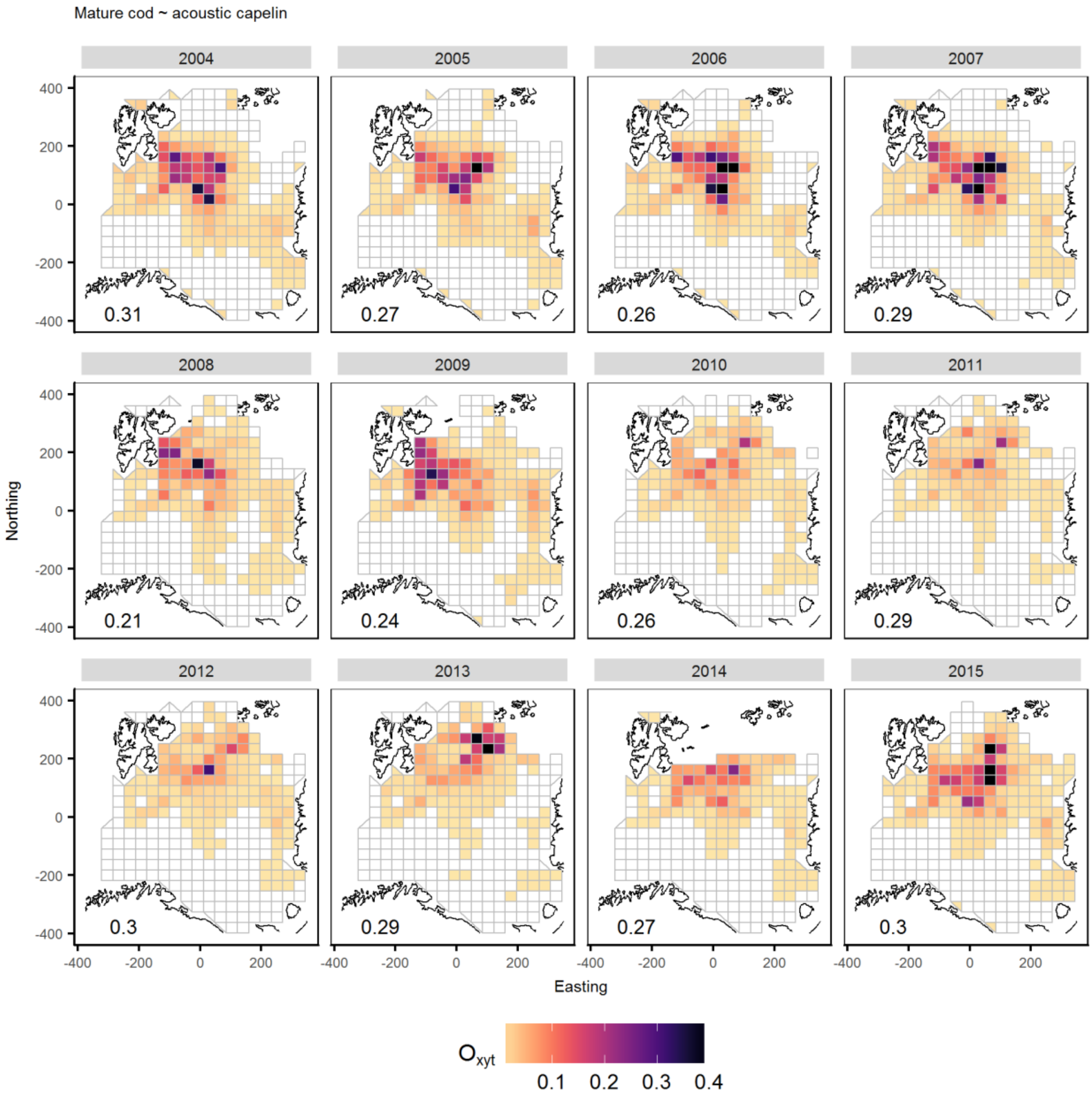

c) Winter: immature cod and acoustic capelin

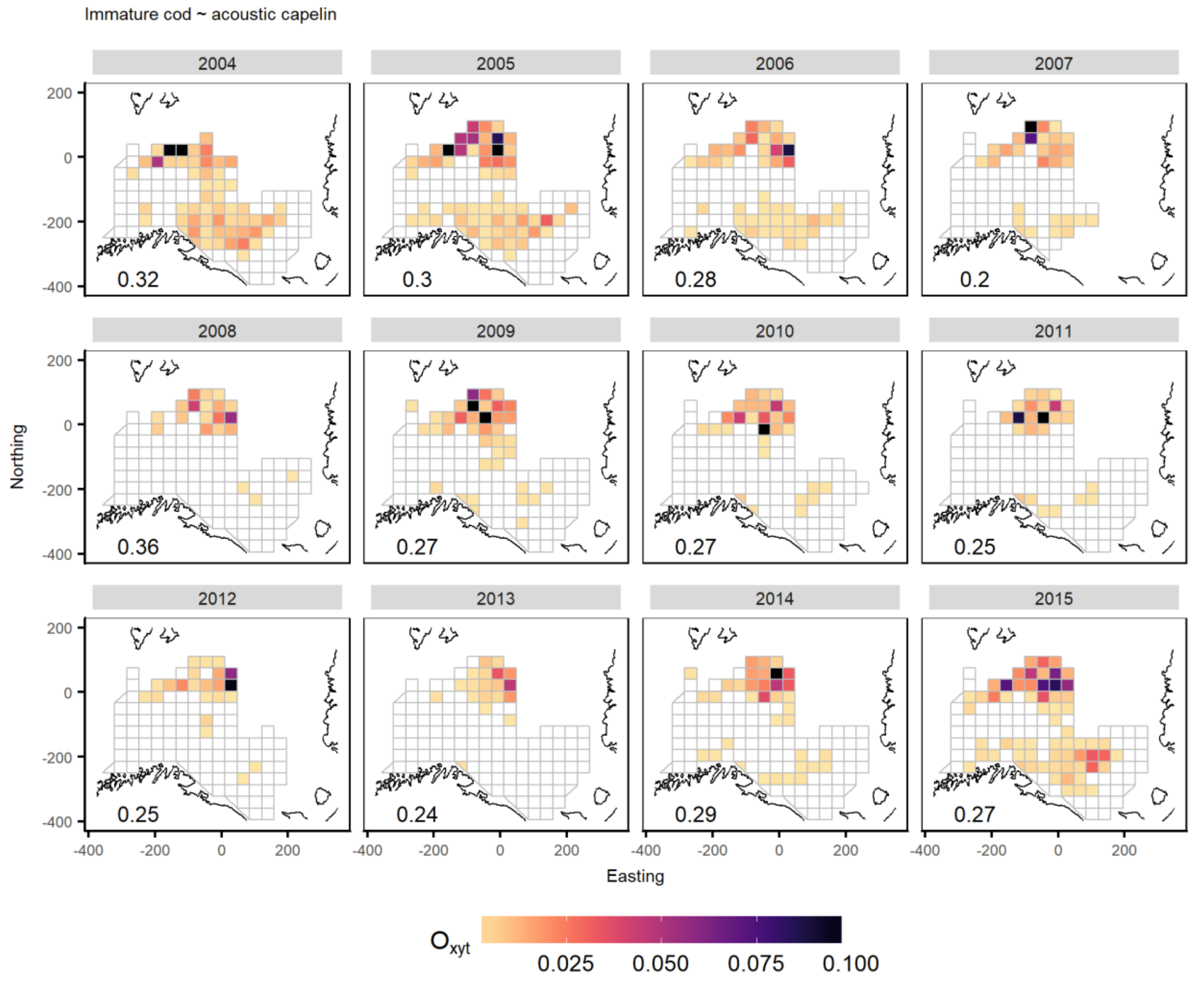

d) Winter: immature cod and trawl capelin

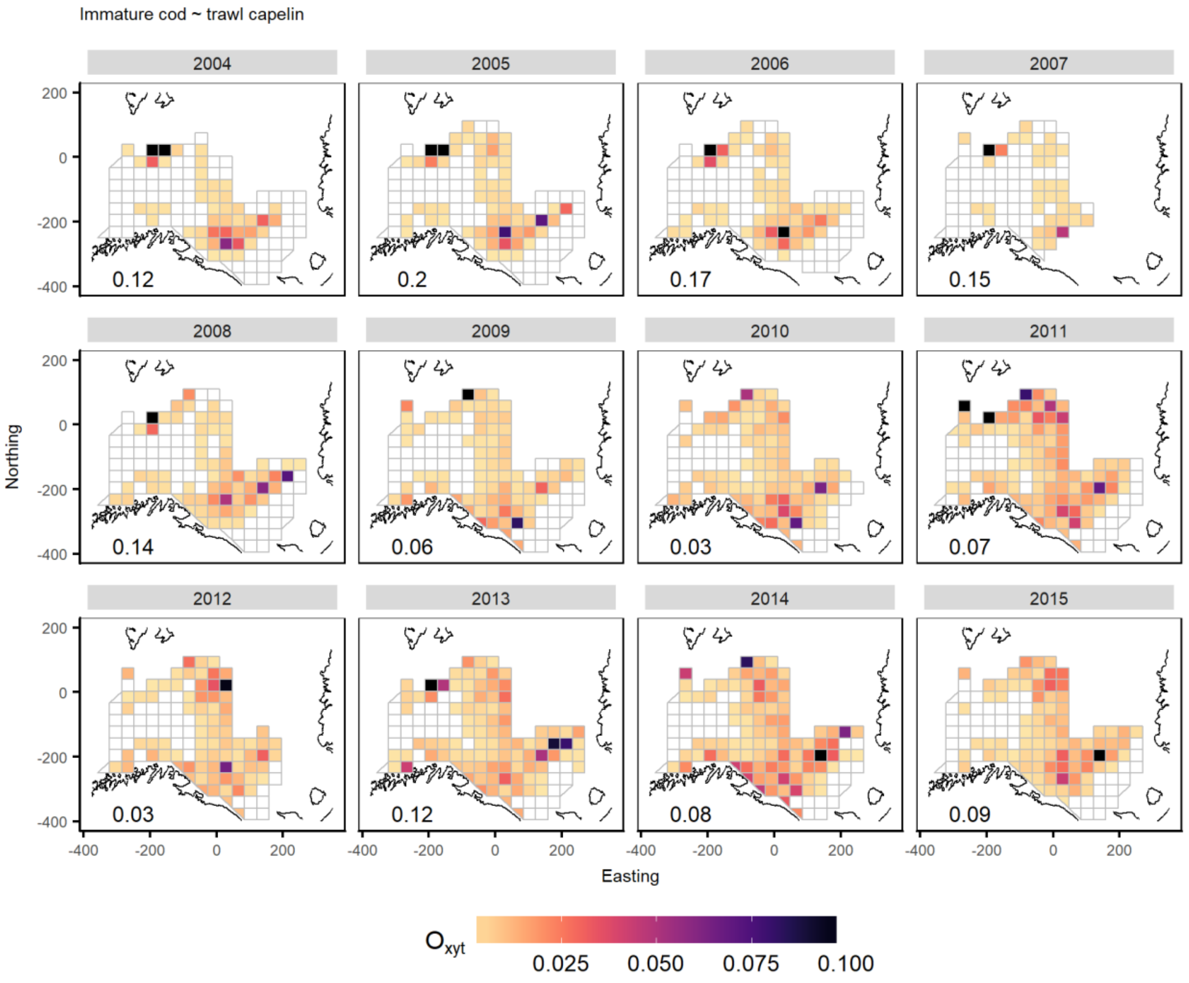

e) Winter: mature cod and acoustic capelin

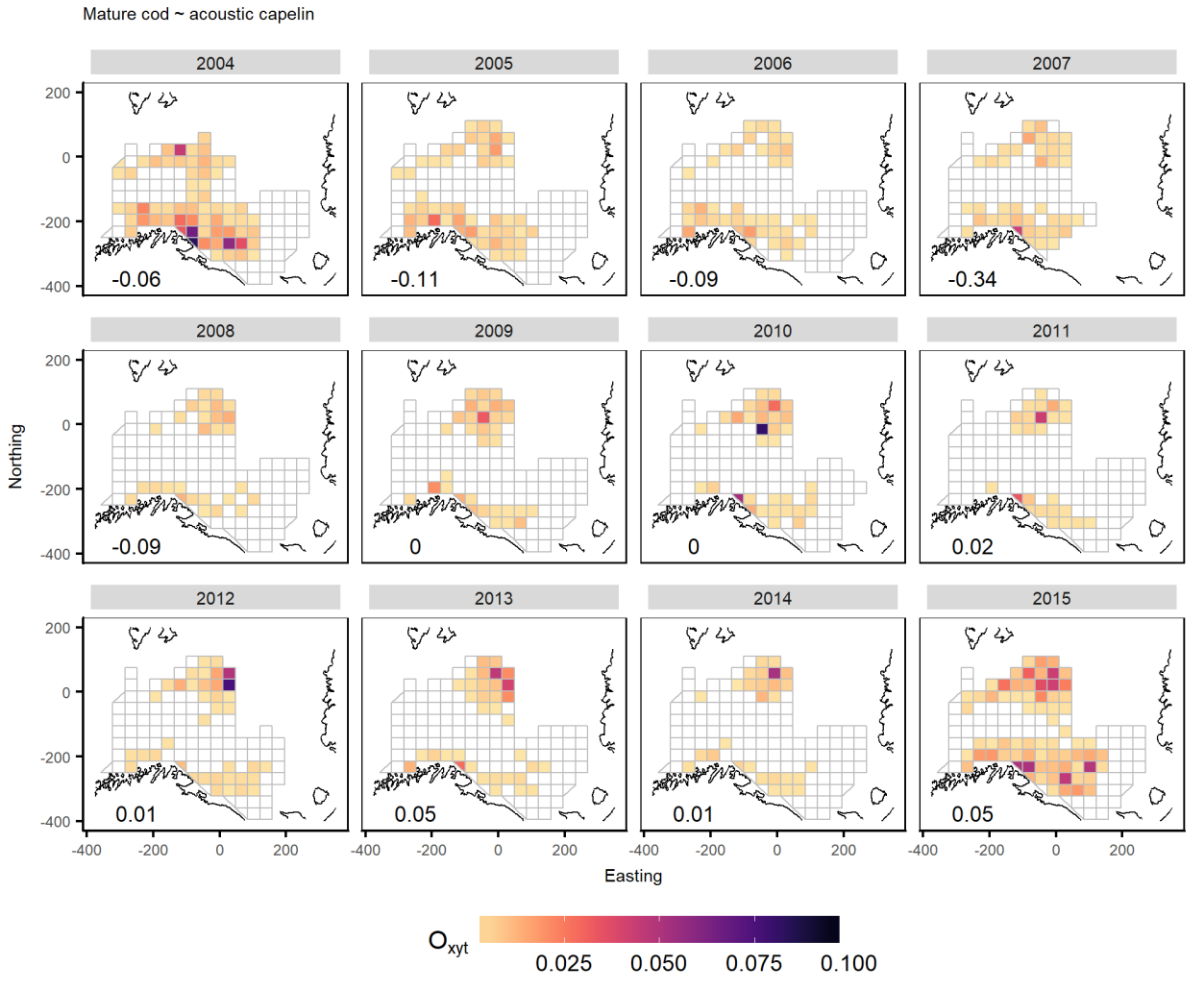

f) Winter: mature cod and trawl capelin

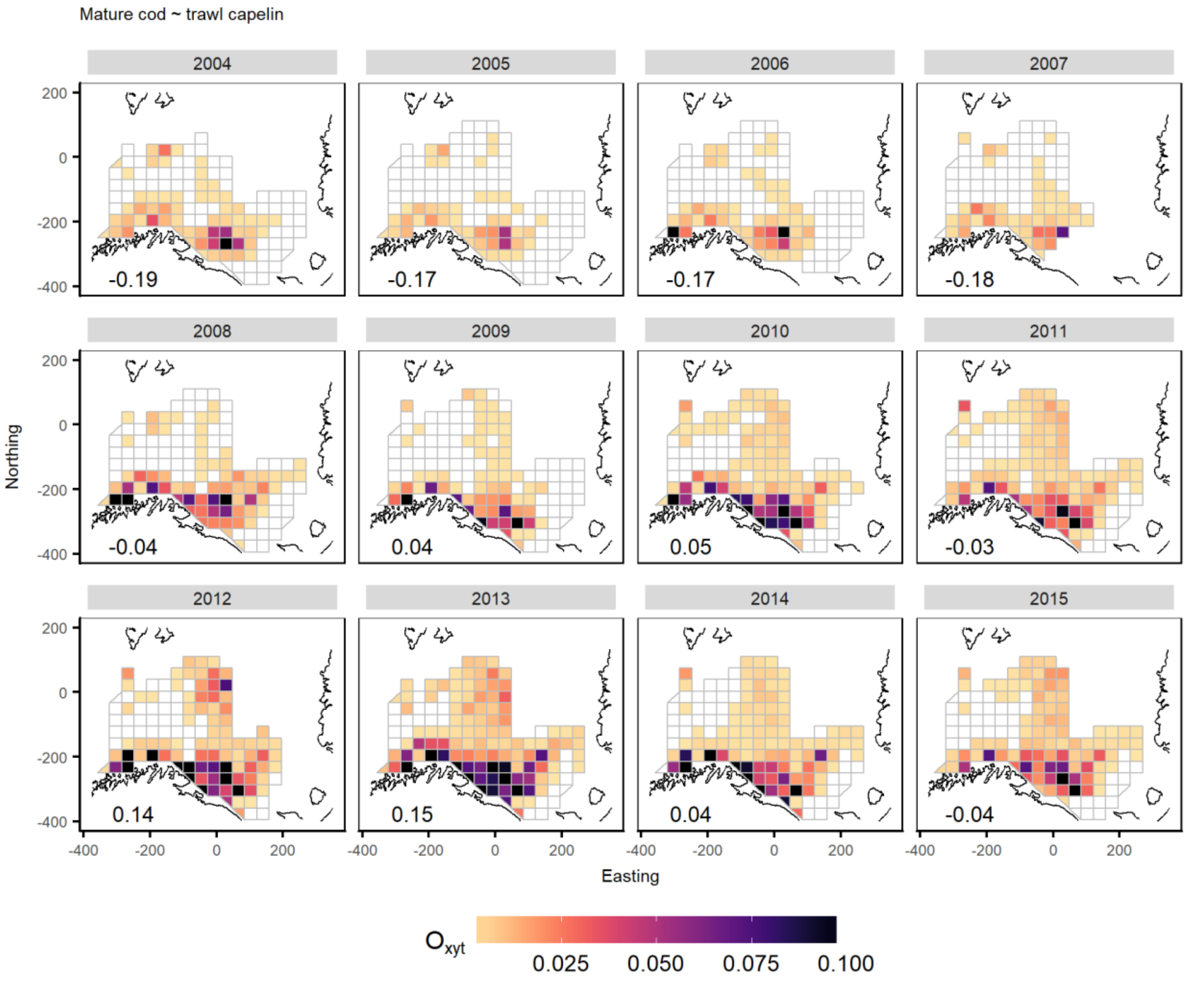

Supplement: S4 Appendix — (PDF) [file pone.0205921.s004.pdf]
